# Genotypic Influences on Actuators of Aerobic Performance in Tactical Athletes

**DOI:** 10.3390/genes15121535

**Published:** 2024-11-28

**Authors:** Martin Flück, Christian Protte, Marie-Noëlle Giraud, Thomas Gsponer, Alain Dössegger

**Affiliations:** 1 Swiss Federal Institute of Sport Magglingen SFISM, 2532 Magglingen, Switzerland; christian.protte@baspo.admin.ch (C.P.); alain.doessegger@baspo.admin.ch (A.D.); 2 Physiogene, 1700 Fribourg, Switzerland; 3 Center for Renal, Hypertensive and Metabolic Disorders, 30625 Hannover, Germany; 4 Cardiology, Department of Endocrinology, Metabolism and Cardiovascular System, University of Fribourg, 1700 Fribourg, Switzerland; marie-noelle.giraud@unifr.ch; 5 STADL Partners, 3110 Münsingen, Switzerland; thomas.gsponer@stadl-partners.ch; 6 Department of Sport, Physical Activity and Health, University of Basel, 4001 Basel, Switzerland

**Keywords:** exercise, fatigue, gene, heart, muscle, resistance, strength, ventilation

## Abstract

Background: This study examines genetic variations in the systemic oxygen transport cascade during exhaustive exercise in physically trained tactical athletes. Research goal: To update the information on the distribution of influence of eleven polymorphisms in ten genes, namely ACE (rs1799752), AGT (rs699), MCT1 (rs1049434), HIF1A (rs11549465), COMT (rs4680), CKM (rs8111989), TNC (rs2104772), PTK2 (rs7460 and rs7843014), ACTN3 (rs1815739), and MSTN (rs1805086)—on the connected steps of oxygen transport during aerobic muscle work. Methods: 251 young, healthy tactical athletes (including 12 females) with a systematic physical training history underwent exercise tests, including standardized endurance running with a 12.6 kg vest. Key endurance performance metrics were assessed using ergospirometry, blood sampling, and near-infrared spectroscopy of knee and ankle extensor muscles. The influence of gene polymorphisms on the above performance metrics was analyzed using Bayesian analysis of variance. Results: Subjects exhibited good aerobic fitness (maximal oxygen uptake (VO_2_max): 4.3 ± 0.6 L min^−1^, peak aerobic power: 3.6 W ± 0.7 W kg^−1^). Energy supply-related gene polymorphisms rs1799752, rs4680, rs1049434, rs7843014, rs11549465, and rs8111989 did not follow the Hardy–Weinberg equilibrium. Polymorphisms in genes that regulate metabolic and contractile features were strongly associated with variability in oxygen transport and metabolism, such as body mass-related VO_2_ (rs7843014, rs2104772), cardiac output (rs7460), total muscle hemoglobin content (rs7460, rs4680), oxygen saturation in exercised muscle (rs1049434), and respiration exchange ratio (rs7843014, rs11549465) at first or secondary ventilatory thresholds or VO_2_max. Moderate influences were found for mass-related power output. Conclusions: The posterior distribution of effects from genetic modulators of aerobic metabolism and muscle contractility mostly confirmed prior opinions in the direction of association. The observed genetic effects of rs4680 and rs1049434 indicate a crucial role of dopamine- and lactate-modulated muscle perfusion and oxygen metabolism during running, suggesting self-selection in Swiss tactical athletes.

## 1. Introduction

Endurance performance is defined as the ability to sustain a physical task for durations ranging from minutes to hours until metabolic and/or central fatigue cause a cessation or reduction in physical work output. This is typically evaluated using an incremental exercise test, where the mechanical output gradually increases from a low intensity until the subject can no longer meet the required power output. Fatigue during prolonged dynamic contractions results from an inability to produce force at the required intensity due to either a contractile insufficiency to produce sufficient power output or an energy deficit in the working muscles [1,2,3]. Thereby, the maximal rate of charging high energy phosphates via aerobic metabolism, as reflected in the ‘maximum oxygen consumption’ during physical exercise, is seen as a critical factor that sets the capacity for the fueling of energetic demand during endurance exercise [4]. 

The delivery of molecular oxygen to muscle tissues is ensured by a systemic transport cascade. This process includes three systemic aspects that have an anatomical basis: (1) ventilation and alveolar gas exchange in the lungs, where oxygen diffuses into pulmonary capillaries and binds to hemoglobin; (2) oxygen transport via the cardiovascular system to muscle tissue capillaries, where it is released from hemoglobin and diffuses into muscle cells; and (3) intracellular oxygen transport, facilitated by myoglobin, and mitochondrial respiration, where oxygen is used to produce adenosine triphosphate (ATP), which fuels muscle contraction [5,6,7,8]. Elements of the oxygen transport cascade all represent actuators of aerobic performance, as they reflect components of the system that converts energy into mechanical motion [9]. The efficiency of oxygen flux through this cascade depends on the capacitive resistance for oxygen transport of the upper and lower airways, alveolea, hemoglobin, heart, capillaries, myoglobin, and mitochondriae [10,11,12]. Variability in systemic oxygen transport capacity can occur due to differences in the functional capacities of these resistive elements, but this remains largely underexplored [13,14].

The oxygen transport cascade serves as a framework to elucidate individual differences in endurance performance based on variations in resistive elements or bottlenecks of aerobic energy supply [11]. Variability in maximal aerobic performance can stem from any part of the oxygen pathway, beyond the capability and efficiency of skeletal muscle phenotypes. Additionally, identical maximal oxygen uptake values could result from different configurations of resistive capacities due to anatomical differences. This has long sparked a debate on the relative contributions of cardiopulmonary versus muscular factors, and lately central-nervous factors, to maximum oxygen uptake (VO_2_max) [15,16]. In this, it may also be worth considering that individual differences in oxygen uptake at submaximal levels, such as the first and second ventilatory thresholds (VT1 and VT2) that mark transitions in oxygen uptake, carbon dioxide exhaustion, and ventilation [17], may also be crucial for training prescription and performance differences during intense efforts such as in competition [18,19].

Genetic polymorphisms that encode metabolic and contractile factors are associated with training-induced variability in muscle performance [20]. Certain polymorphisms determine the structure and functionality of enzymes and functional proteins at the cellular level. This is why gene variants can be considered a partial cause of inter-individual performance variability. This is true in all performance-related structures, especially in the oxygen transport cascade. Single gene variants can affect aerobic performance and capacity by up to 20% [20,21,22,23]. Approximately 200 gene polymorphisms are associated with differences in physical performance, often interacting in complex ways. A meta-analysis found that genetic factors explain 44% of the variance in cardiovascular fitness, 72% in muscular strength, and 10% in anaerobic power responses to physical training [24].

Prominent gene polymorphisms affecting the oxygen pathway through varied mechanisms include rs699 and rs1799752 in the functionally connected genes for angiotensinogen (AGT) and angiotensin-converting enzyme (ACE), which control cardiovascular supply. ACE mediates the proteolytic cleavage of the deca-peptide angiotensin 1, that results from the processing of the liver-derived large AGT precursor, to produce the angiotensin 2 peptide that exerts myogenic effects on smooth and cardiac muscle [25]. As such, angiotensin 2 levels influence blood vessel density and perfusion, cardiac muscle anatomy, and stroke volume [26]. The latter aspects of cardiovascular functioning are influenced by the aforementioned polymorphisms through increased levels of AGT in consequence of the exchange of methionine for threonine at amino acid position 235 (rs699) and increased ACE expression in deletion allele carriers of rs1799752, respectively [27,28,29]. The association of gene polymorphism rs1799752 with aerobic and anaerobic capacity henceforth is linked to its effects on angiotensin 2, impacting cardiovascular and muscular energy provision during intense exercise, and affecting the functional response to physical training via influences on left ventricular hypertrophy, angiogenesis, mitochondrial biogenesis, and strength gains in muscle groups [21,28,29,30,31]. Polymorphisms rs2104772 in the tenascin-C gene and rs4680 in the catechol-O-methyltransferase (COMT) gene are reported to affect mitochondrial metabolism in muscle tissue and brain, respectively, through influences on the volume density and perfusion of capillaries [32,33,34,35,36,37,38,39,40]. The influence of rs2104772 on capillary growth is mediated by the non-synonymous exchange of amino acids thymidine (T)-to-adenosine (A) in codon 1677 of tenascin-C that affects endurance exercise-induced angiogenesis in skeletal muscle [34]. Analogously, polymorphism rs4680 describes the substitution of valine for methionine in amino acid codon 158 of COMT due to the exchange of guanidine (G) for adenosine (A), which is associated with lowered oxygen-dependent metabolism in the forebrain during intense cognitive tasks [41]. The underpinning mechanism involves increased levels of the dopamine hormone, which acts as a vasoconstrictor and integrated regulator of motor physiology that controls skeletal muscle tone and, in consequence, influences muscle perfusion [42]. rs4680 is associated with differences in the degree of improvements in cognitive performance in young adults with physical training [43] and personality traits in combat athletes and controls [44]. Despite preponderant indications for the association of rs4680 with aerobic metabolism and cardiovascular perfusion [45], no single report so far has addressed the role of rs4680 in setting physiological characteristics of aerobic or anerobic performance [46]. Additionally, rs7460 and rs7843014 in the gene for focal adhesion kinase (PTK2) impact the mechano-biology of cardiac muscle, the cardiovascular system, and skeletal muscle [47,48,49,50]. Both polymorphisms affect the expression levels and activity of PTK2 [51], which affect mechano-regulated protein synthesis and stiffness in skeletal muscle [52,53] and connect to differences in mechano-regulated morphogenesis of blood vessels and myocardial remodeling [54,55,56]. Polymorphisms rs11549465 and rs1049434 in the genes for hypoxia-inducible factor 1 alpha (HIF1A) and monocarboxylate transporter 1 (MCT1) genes are linked to variations in aerobic metabolism and endurance performance via influences on aerobic metabolism [29,57]. rs11549465 describes a non-synonymous A-to-T exchange in MCT1 that lowers the transmembrane export of lactate and affects the utilization of metabolic substrates during intense exercise [29]. The altered stability of the transcription factor HIF1A due to the exchange of cytosine for thymidine and the subsequent replacement of proline by serine of amino acid position 582 modulates the expression of metabolic enzymes and angiogenic factors [58,59,60]. Further on, polymorphism rs1815739 in the ACTN3 gene influences the contractility and metabolic efficiency of skeletal muscle [58,59,60] by modulating the stability of fast--type muscle fibers. Thymidine carriers produce a truncated cytoskeletal actinin-3 protein compared to cytosine carriers [61], which is tied to the destabilization of the cytoskeleton in fast-type muscle fibers [62]. Moreover, the myostatin gene (MSTN) has been found to affect muscle fatigue resistance and aerobic capacity in mice [63], and the MSTN polymorphism rs1805086 is understood to affect muscle power [64,65]. Finally, rs8111989 in the creatine kinase (CKM) gene affects the replenishment of metabolic resources during strenuous exercise through its action in shuttling ATP from the sites of ATP production (glycolysis and mitochondrial oxidative phosphorylation) with subcellular sites of ATP utilization [66,67,68].

Tactical athletes, such as special operations police and army personnel, are selected and trained for their ability to perform highly strenuous tasks over extended periods. These individuals require high stamina to handle physically demanding tasks while managing heavy equipment. Consequently, they must exhibit significant fatigue resistance at exercise intensities and muscle loading near or above their maximum aerobic capacity [69,70]. Talent, which may reflect genetic factors to a certain degree, is the unfolding of the full physical potential with years of physical training and may indicate venues for tailoring interventions (including diet and exercise) to individual constitutional capacities to maximize athletic potential [26,57,71,72,73,74,75]. However, published data on the genetic profile of tactical athletes are extremely sparse. We could only identify three groups that published data. Indian special forces (paratroopers) were statistically significant different from non-paratroopers with regard to the ACTN3 polymorphisms (31.3% vs. 20.0% RR alleles) [76]. Boscarino et al. (2022) published associations between, among others, the COMT polymorphisms and suicidal ideation; however, they did not report the distribution of the alleles among their sample of veterans [77]. In US Navy Explosive Ordnance Disposal (EOD) operators, genetic variants underlying human stress systems (5HTTLPR, BclI, −2 C/G, and COMT) were assayed. Serotonin transporter gene 5HTTLPR consistently predicted CRF and/or aerobic performance [78].

**Figure 1:**
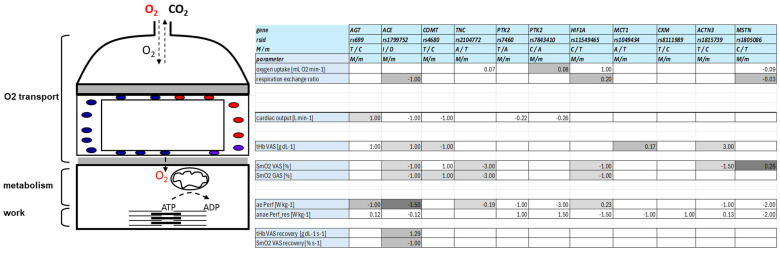
Visualized hypotheses. Composite drawing of the research hypothesis of genetic influences on oxygen transport and aerobic performance. (**Left**) Assessed elements of the oxygen transport cascade during muscle work. (**Right**) Color-coded listing of the hypothesized effects of gene polymorphism on the assessed parameters of the oxygen transport cascade (prior knowledge). Numbers denote the mean-centered differences (step size) between major and minor alleles for the hypothesized effects of a respective gene polymorphism. Light gray cells indicate hypothesized influences of the intensity of exercise or prior exercise/warm-up. Darkly highlighted cells denote those where the prior available information was contradictory. Empty cells denote instances where no prior information was indicated to formulate a specific hypothesis. Abbreviations: ACE, angiotensin-converting enzyme; ACTN3, alpha actinin-3; ADP, adenosine diphosphate; aePerf, aerobic performance; AGT, angiotensinogen; ATP, adenosine triphosphate; anae Perf_res, anaerobic power reserve; CKM, muscle-type creatine kinase; CO_2_, carbon dioxide; COMT, catechol-O-methyltransferase; GAS, gastrocnemius (medialis) muscle; HIF1A, hypoxia-inducible factor 1 alpha; m, minor variant of a gene polymorphism; M, major variant of gene polymorphism; MCT1, monocarboxylate transporter 1; MSTN, myostatin; PTK2, protein tyrosine kinase 2 (or focal adhesion kinase); O_2_, oxygen; rsid, identifier of gene polymorphism; SmO_2_, muscle oxygen saturation; TNC, tenascin-C; tHb, total hemoglobin concentration; VAS, vastus lateralis muscle.

This investigation aimed at conducting an exploratory analysis to update information from previous research on the influence of eleven gene polymorphisms on aerobic metabolism and performance during endurance exercise. We therefore focused on possible sites of influences of the selected genetic variants on proxy variables for the main steps of the oxygen transport cascade (as depicted in Figure 1) [24]. A Bayesian-based statistical approach [79,80] was conducted, as this allows for testing the probability of the alternative relative to the null hypothesis. This renders this method also more flexible for hypothesis testing and allows for updating statistical assumptions on priors with information once they become available. Further, this study explored possible interactions of different gene polymorphisms on aerobic and anaerobically physical performance.

## 2. Materials and Methods

Ethics: This study was performed in accordance with current ethical guidelines (Declaration of Helsinki, as revised in 2013) and was approved by the Human Research Ethics Committee of the Canton of Berne (no. 2022-00767), as well as the research committee of the Swiss Armed Forces (Forschungsausschuss San D Armee, Kompetenzentrum Militär und Katastrophenmedizin, 2022-00767), approved date 10 May 2022.

Subjects: Study participants were recruited from currently active military and police SOF operators and SOF candidates, both from professional and militia units. Participants were pseudonymized through a study ID by their employers or the recruiting authority. 

Graded cardiopulmonary exercise test into exhaustion: Loaded, graded cardiopulmonary exercise testing (CPET) on a treadmill (h/p Cosmos Pulsar, Nussdorf-Traunstein, Germany) was embedded in a fitness test battery that included, in sequential order, anthropometry, reaction time, strength, and power test. Subjects, wearing light sports clothes and sneakers and a weight vest (12.6 kg), rested for 15 min before running. They were equipped with a heart rate sensor (Polar H10, Polar Oy, Kempele, Finland) attached to a chest-worn belt and two near-infrared spectroscopy devices (Moxy, Fortiori Design LLC., Hutchinson, MN, USA) placed on the knee and ankle extensor muscle, *m. vastus lateralis*, and *m. gastrocnemius*. A calibrated breath-by-breath spirometry system (MetaMax 3B-R2, Cortex Biophysics, Leipzig, Germany) was used to quantify expired gases as collected through a Hans Rudolf facemask. Cardiopulmonary exercise began with a warm-up at a 0° incline at 5.5 km/h. During the test phase, the treadmill was set to 8 km/h with an increase in the incline by 0.1° every 6 s until subjects could no longer maintain the speed. Measurements continued during the recovery phase while subjects were asked to sit on a chair on the treadmill. Lactate concentration was measured using a Lactate Photometer Plus DP 110 (Diaglobal, Berlin, Germany) in 10 μL of capillary blood from the earlobe, collected immediately and 5 min after the run. Training habits were collected via questionnaire. All measurements were performed in the time period from February 2022 until October 2023.

Genotyping: Buccal swaps were collected in a preserving solution (DNA/RNA Shield Collection Tube w/Swab, R1109, Zymo Research, Distributor Lucerna-Chem AG, Lucerne, Switzerland) and kept at a temperature between 4 and 8 °C for up to one month before being processed. Genomic DNA was extracted using a DNeasy blood and tissue kit (Cat No. 69504. Qiagen, Hombrechtikon, Switzerland). Amplicons containing the nucleotide polymorphism of interest were identified using 200 nM sense and antisense oligonucleotides in reaction mixes with the DNA polymerase, i.e., KAPA SYBR FAST Mix (KK4603, Kapa Biosystems, ABI Prism, Merck, Buchs, Switzerland) or SensiFAST HRM Mix (Meridian Biosciences; BIO32005, Labgene, Châtel-St-Denis, Switzerland), with temperature protocols on a magnetic induction cycler (Mic Real-Time PCR System, Bio Molecular Systems; Labgene, Châtel-St-Denis, Switzerland) as outlined in Table 1. Reactions were analyzed for variations in the detection cycle and high-resolution melting (HRM) curves, using genotype references established in prior PCR experiments. These references were based on Sanger sequencing of amplicons from selected PCR reactions, utilizing specific oligonucleotide primers provided by Microsynth (Balgach, Switzerland). Additionally, PCR amplicons for which the confidence level for HRM-based genotyping was lower were verified with Sanger sequencing.

Data handling and calculations: Data were collected and managed using REDCap electronic data capture tools (v13.7.3) [81] hosted at the Federal Office of Sport (FOSPO), Magglingen, Switzerland. All data were entered using the pseudonym of the subjects as ID. The CPET data were synchronized and used to derive and estimate parameters of interest, such as proxy values for systemic aspects of oxygen transport at the different ventilatory thresholds (VT) that were used to characterize the intensity of exercise, i.e., start, VT1, VT2, VO_2_max. VT were determined according to published criteria [82,83]. 

The measured proxy response variables of oxygen transport were for ventilation and alveolar gas exchange in the lungs: VO_2_ at VT1, VO_2_ at VT2, VO_2_max, as quantified by spiroergometry; for oxygen transport via the cardiovascular system to muscle tissue capillaries: cardiac output (Q′), total hemoglobin concentration (tHb), and SmO_2_ recovery as quantified by ergospirometry and NIRS; for intracellular oxygen transport and mitochondrial respiration: SmO_2_, respiratory exchange ratio (RER), and lactate concentration. Proxy variables of global performance were derived from the computed power output and anaerobic power reserve (both in W/kg body mass). The values for power output corresponded to those automatically generated from the Metasoft software (version 1.04.18), which was calculated from a formula (1.065 + 0.0511 × incline [%] + 9.322 × 0.0001 × incline [%] × incline [%]) × velocity × BM/4. The calculation of the anaerobic power reserve was based on the difference between the observed peak power output and the maximal aerobic power (at VT2) during the running test to exhaustion [84,85]. 

Cases with missing values were excluded from the analyses. Missing values were exclusively due to technical malfunction of the equipment deployed. Data were examined for outliers using their z-scores. Notable values were checked for recording errors and corrected if necessary. If a z-score exceeded 6, the corresponding data point was removed from the dataset before statistical analysis. This concerned three values for tHb only. 

Statistics: Data were descriptively assessed for mean, standard deviation (SD), and standard error (SE), as well as maximal (max) and minimal (min) values for each response variable. Data were displayed in line plots or box plots and superimposed individual values. Hardy–Weinberg equilibrium was assessed using an Excel-based calculation of the genotype distribution with a Chi2-test as described [86].

The effect of a gene polymorphism on f the response variable was evaluated by Bayesian (repeated measures) analysis of variance (ANOVA) using the software JASP (vs. 0.18.3.0, University of Amsterdam, https://jasp-stats.org/download/ (accessed on 9 January 2024); https://jasp-stats.org/jasp-materials/ (accessed on 23 November 2024)) [87]. We compared two models, one model where the genotypes of the assessed gene polymorphisms have an additive impact on individual physiological factors (alternative hypothesis model) and one model without genotypes (null model). For repeated measures, the exercise intensity at the start, VT1, VT2, and VO_2_max were defined as repeated factors. The distribution of the residuals was assessed descriptively in histograms, Q-Q plots, and based on the Shapiro–Wilk test. For further information, see Supplemental Figures S1 and S2, and Table S1. We modeled our weak prior knowledge with r-scales of 0.5 for fixed and 1 for random effects, respectively. Models were compared using the Bayes factor (BF10), which indicates evidence for the likelihood of the data under the alternative model relative to the data under the null model. BF10 was categorized into four intervals for numbers above 1: 3 (moderate), 10 (strong), 30 (very strong), and 100 (extreme support in favor of the alternative model), following previous recommendations [80], allowing for quantifying continuous measures of support. BF10 can be regarded as an odds ratio that quantifies the change in belief from prior odds to posterior odds [88]. Post hoc pairwise comparison was conducted using Bayesian *t*-tests if BF10 was greater than a rounded value of 3.0, i.e., >2.5.

## 3. Results

### 3.1. Physiological Characteristics of the Test of Loaded Ramped Running Exercise

Table 2 details the functional characteristics of the 251 assessed subjects and their training type. Most subjects demonstrated good aerobic capacity with values for VO_2_max above 50 mL min^−1^ kg^−1^ [83]. Considering German police SOF (52.4 ± 4.1 mL min^−1^ kg^−1^) [89] or British Special Air Service (55.00 ± 5.20 mL min^−1^ kg^−1^, [90]), the mean of 53.23 ± 7.33 mL min^−1^ kg^−1^ of our Swiss tactical athletes was comparably high.

**Figure 2:**
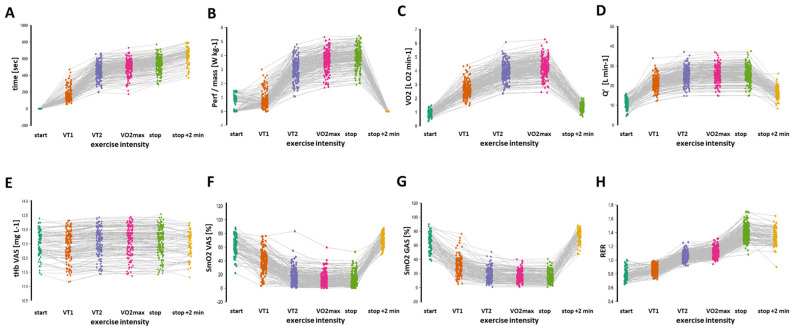
Physiological characteristics during loaded, graded exercise. Rain cloud line plots of individual values for assessed characteristics of oxygen transport (Y-axes) during the different phases (X-axes) of loaded and graded running exercise into exhaustion for the studied 251 subjects. Cases with missing data for a phase were excluded from the display. (**A**–**H**) time (**A**), performance (**B**), VO_2_ (**C**), Q′ (**D**), tHb in VAS (**E**), SmO_2_ in VAS (**F**), SmO_2_ in GAS (**G**), and RER (**H**). Abbreviations: max, maximal values; stop + 2 min, 120 s into recovery after the cessation of running; VO_2_, oxygen uptake; VT1, ventilatory threshold 1; VT2, ventilatory threshold 2; Perf, power; Q′, cardiac output.

Figure 2 shows the progression of mechanical and metabolic performance during the exercise test at VT1 and VT2, VO_2_max, and the first 2.5 min of passive recovery. Compared to values at the start, average oxygen uptake increased approximately four-fold until VO_2_max, while SmO_2_ in knee and ankle extensor muscles decreased about four times. Q′ increased on average 2.3 times at VO_2_max, with tHb rising by roughly 1%. RER and blood lactate concentration at the end of the run reached 1.38 ± 0.01 and 13.69 ± 0.21 mM, respectively, indicating a significant contribution of anaerobic metabolism towards exhaustion. After stopping the running, SmO_2_, VO_2_, and Q′ returned to near pre-exercise levels within approximately 2 min.

### 3.2. Genetic Characteristics

The overall genotype distribution is presented in Table 3. A departure from the Hardy–Weinberg distribution was observed for gene polymorphisms rs1799752 (ACE), rs4680 (COMT), rs1049434 (MCT1), rs7843014 (PTK2), rs11549465 (HIF1A), and rs8111989 (CKM).

### 3.3. Exploratory Associations of Genotypes with Oxygen Transport

Table 4 summarizes the overall BF10 of the models, including the studied gene polymorphisms as factors to explain the variance of the response variables of the oxygen transport cascade during loaded ramped running to exhaustion and subsequent recovery. Gene polymorphisms showed varied influences on pulmonary, cardiovascular, and muscle metabolic variables of oxygen transport. Exercise intensity had a decisive overall influence on the variability of all assessed response variables.

### 3.4. Exploratory Influence on Pulmonary Parameters

**Figure 3:**
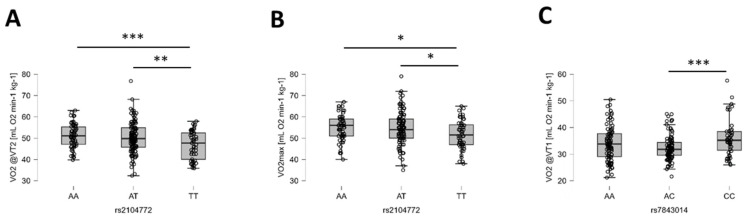
Examples of the identified genotype effects on body mass-related oxygen uptake. (**A**–**C**) Box plots (line: median; cross: mean; box: data from first to third quartile; whiskers: ±1.5 × interquartile range) and individual values (circles) for the influence of rs2104772 on VO_2_ at VT2 (**A**) and VO_2_max (**B**), and rs7843014 at VT1 (**C**). Y-axes resume the identity of the respective response variable and applicable unit, while the X-axes indicate the respective genotypes of the addressed gene polymorphism. Respective Bayes factors (BF10) for post hoc effects are given as follows: *, 10.0 ≥ BF10 > 2.5; **, 30.0 ≥ BF10 > 10.0; ***, BF10 > 30.0. Abbreviations: VO2@VT1, VO_2_ at VT1; VO2@VT2, VO_2_ at VT2.

Based on the evidence formulation of BF10, the alternative hypothesis of a genetic influence on pulmonary performance during ramped loaded running to exhaustion was probable for two gene polymorphisms, rs2104772 and rs7843014 (Table 4). For body mass-related VO_2_, there were strong probable influences of rs2104772 (TNC) at VT2 and rs7843014 (PTK2) at VT1, which were identified through a post hoc analysis (Figure 3).

### 3.5. Exploratory Influence on Cardiovascular Parameters

**Figure 4:**
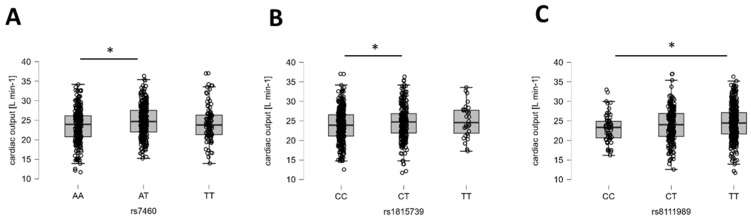
Examples of the identified genotype effects on cardiac output. (**A**–**C**) Box plots (line: median; cross: mean; box: data from first to third quartile; whiskers: ±1.5 × interquartile range) with individual values (circles) for the influence of rs7460 (**A**), rs1815739 (**B**), and rs8111989 (**C**) on overall cardiac output. Y- and X-axes resume the identity of the respective response variable, the applicable unit, and the respective genotype. Respective Bayes factors (BF10) for post hoc effects are given as follows: *, 10.0 ≥ BF10 > 2.5.

Based on the evidence formulation, BF10, cardiac output showed evidence for being influenced by rs7460 (PTK2), with moderate influences from rs8111989 and rs1815739 (Table 4), which were identified through post hoc analysis (Figure 4).

**Figure 5:**
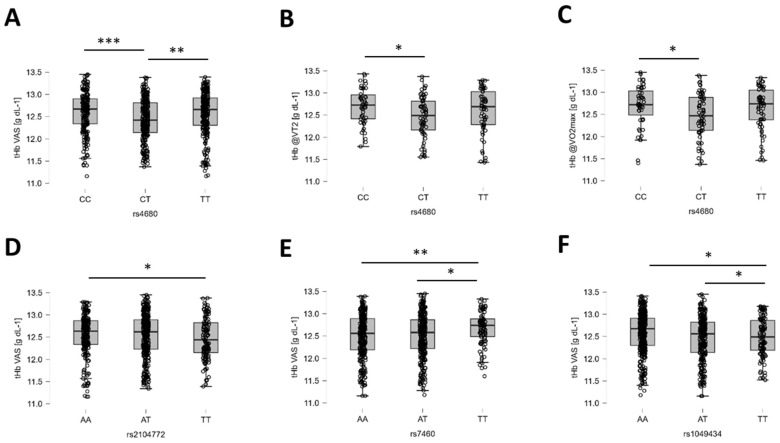
Examples of the identified genotype effects on total hemoglobin concentration of vastus lateralis muscle. (**A**–**F**) Box plots (line: median; cross: mean; box: data from first to third quartile; whiskers: ±1.5 × interquartile range) with individual values (circles) for the influence of rs4680 on overall tHb in VAS (**A**), tHb in VAS at VT2 (**B**), and tHb in VAS at VO_2_max (**C**), as well as rs2104772 (**D**), rs7460 (**E**), and rs1049413 (**F**) on overall tHb in VAS. Y- and X-axes resume the identity of the respective response variable, the applicable unit, and the respective genotype. Respective Bayes factors (BF10) for post hoc effects are given as follows: *, 10.0 ≥ BF10 > 2.5: **, 30.0 ≥ BF10 > 10.0; ***, BF10 > 30.0. Abbreviations: tHb@VT2, tHb at VT2; tHb@VO2max, tHb at VO_2_max.

Total hemoglobin concentration in the vastus lateralis muscle demonstrated a strong probability of association with rs4680 and rs7460 and moderate probabilities of influence from rs2104772 and rs1049434 (Table 4). For rs4680, this was localized to moderate probabilities for post hoc differences at VT2 and VO_2_max (Figure 5).

### 3.6. Exploratory Influence on Muscle Metabolism Related Parameters

**Figure 6:**
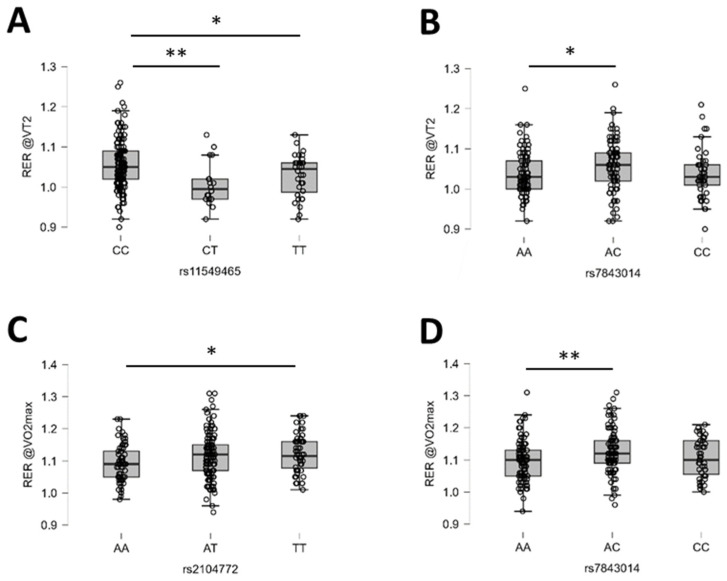
Examples of the identified genotype effects on respiration exchange ratio. (**A**–**D**) Box plots (line: median; cross: mean; box: data from first to third quartile; whiskers: ±1.5 × interquartile range) with individual values (circles) for the influence of rs11549465 (**A**) and rs7843014 (**B**) on RER at VT2, and rs2104772 (**C**) and rs7843014 (**D**) on RER at VO_2_max. Y- and X-axes resume the identity of the response variable, the applicable unit, and the respective genotype. Respective Bayes factors (BF10) for post hoc effects are given as follows: *, 10.0 ≥ BF10 > 2.5; **, 30.0 ≥ BF10 > 10.0. Abbreviations: RER@VO2max; RER at VO_2_max; RER@VT2; RER at VT2.

The evidence formulation of BF10 identified moderate-to-very-strong genotype influences for aspects of aerobic muscle metabolism, such as SmO_2_ and RER, which reflect the metabolic activity of specific muscles and overall engaged organs during exercise (Table 4). Respiratory exchange ratio showed a strong probable effect of rs7843014 (PTK2) and rs11549465 (HIF1A), with a moderate probability of an effect from rs2104772 (TNC; Table 4). Figure 6 illustrates the identified post hoc differences for RER.

The probable influence on oxygen saturation in skeletal muscles was identified for five gene polymorphisms: rs4680, rs7460, rs11549465, rs1049434, and rs1815739. Oxygen saturation of the knee extensor muscle (m. vastus lateralis) and the medial portion of the ankle extensor muscle (m. gastrocnemius medialis) showed the strongest probability of influence on rs1049434 (MCT1) before the start of running exercise (Table 4). During graded running exercise, a strong probability of effect for rs1049434 (MCT1) was identified at VT1 for *m. vastus lateralis* and a moderate probability of effect at VO_2_max for *m. gastrocnemius*.

**Figure 7:**
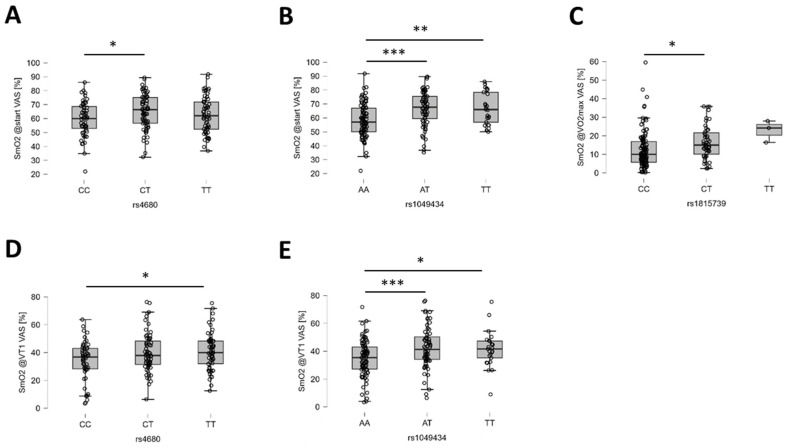
Examples of the identified genotype effects on oxygen saturation in vastus lateralis muscle. (**A**–**E**) Box plots (line: median; cross: mean; box: data from first to third quartile; whiskers: ±1.5 × interquartile range) with individual values (circles) for the influence of rs4680 (**A**) and rs1049434 (**B**) on SmO_2_ in VAS at the start of exercise, as well as rs1815739 on SmO_2_ in VAS at VO_2_max (**C**), and rs4680 (**D**) and rs1049434 (**E**) SmO_2_ in VAS at VT1. Y- and X-axes resume the identity of the response variable and the applicable unit, and the respective genotype. Respective Bayes factors (BF10) for post hoc effects are given as follows: *, 10.0 ≥ BF10 > 2.5; **, 30.0 ≥ BF10 > 10.0; ***, BF10 > 30.0. Abbreviations: SmO2@start, SmO_2_ at start VAS; SmO2@VO2max, SmO_2_ at VO_2_max; SmO2@VT1, SmO_2_ at VT1.

**Figure 8:**
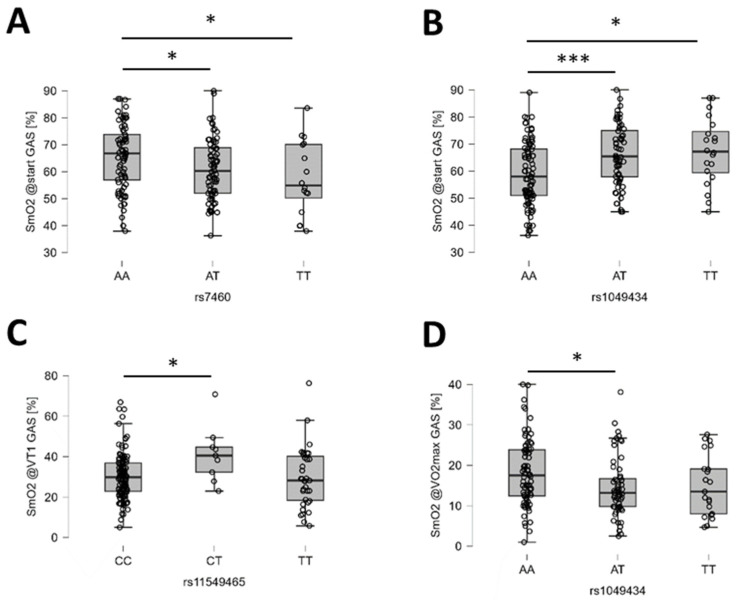
Examples of the identified genotype effects on oxygen saturation in gastrocnemius medialis muscle. (**A**–**D**) Box plots (line: median; cross: mean; box: data from first to third quartile; whiskers: ±1.5 × interquartile range) with individual values (circles) for the influence of rs7460 (**A**) and rs1049434 (**B**) on SmO_2_ in GAS at the start of exercise, as well as rs11549465 on SmO_2_ in GAS at VT1 (**C**) and rs1049434 on SmO_2_ in GAS at VO_2_max (**D**). Respective Bayes factors (BF10) for post hoc effects are given as follows: *, 10.0 ≥ BF10 > 2.5; ***, BF10 > 30.0.

Oxygen saturation of the vastus lateralis muscle at rest and VT1 also demonstrated a moderate probability of association with rs4680 (COMT). At VO_2_max, SmO_2_ of *m. vastus lateralis* showed a moderate probability of association with rs1815739 (ACTN3). For *m. gastrocnemius*, SmO_2_ at rest and VT1 demonstrated moderate probabilities of association with rs7460 (PTK2) and rs11549465 (HIF1A), respectively. Figure 7 and Figure 8 illustrate the identified post hoc differences in SmO_2_ in *m. vastus lateralis* and *m. gastrocnemius* during ramp-loaded exercise.

### 3.7. Exploratory Influence on Physical Performance

**Figure 9:**
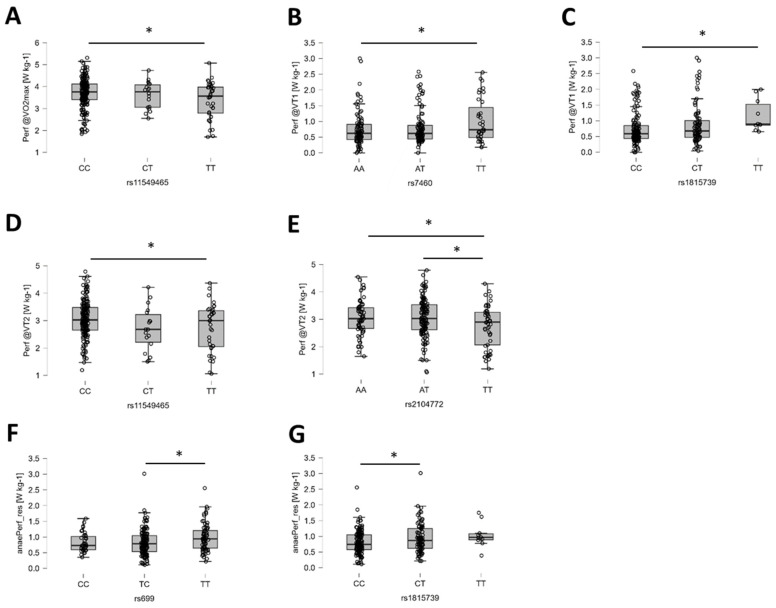
Examples of the identified genotype effects on power output. (**A**–**G**) Box plots (line: median; cross: mean; box: data from first to third quartile; whiskers: ±1.5 × interquartile range) with individual values (circles) for the influence of rs11549465 on performance at VO_2_max (**A**), rs7460 (**B**), and rs1815739 (**C**) on performance at VT1, as well as rs11549465 (**D**) and rs2104772 (**E**) on performance at VT2, as well as rs699 (**F**) and rs1815739 (**G**) on the anaerobic power reserve. Y- and X-axes resume the identity of the response variable, the applicable unit, and the respective genotype. Respective Bayes factors (BF10) for post hoc effects are given as follows: *, 10.0 ≥ BF10 > 2.5. Abbreviations: Perf@VO2max, power at VO_2_max; Perf@VT1, power at VT1; Perf@VT2, power at VT2.

The alternative hypothesis of an influence on power output at VT1, VT2, or VO_2_max was moderately more probable for four gene polymorphisms, i.e., rs2104772 (TNC), rs7460 (PTK2), rs11549465 (HIF1A), and rs1815739 (ACTN3; Table 4). For the anaerobic power reserve, this concerned rs699 (AGT) and rs1815739 (ACTN3). Figure 9 visualizes the identified post hoc difference for muscle performance. Moderate probabilities of an effect on the anaerobic power reserve were noted for the studied gene polymorphisms of AGT (rs699) and ACTN3 (rs1815739). 

### 3.8. Exploratory Influence on Recovery from Exercise

Recovery in total hemoglobin concentration and oxygen saturation of *m. vastus lateralis* after exhaustive loaded and graded running exercise persisted for rs1815739 and rs4680, respectively, into the first two minutes of recovery when a strong probability of effect was identified for rs1799752 (Table 4, Figure S3). 

SmO_2_ in *m. vastus lateralis* and *m. gastrocnemius medialis* during the first two minutes of recovery did not demonstrate probable influences for any of the studied gene polymorphisms.

Gross anatomical influences: From the anthropometric factors, body mass alone demonstrated a moderate-to-strong influence of PTK2-assoicated gene polymorphisms, i.e., BF10 values of 3.6 and 26.1 for rs7460 and rs7843014, respectively. 

## 4. Discussion

While the influence of genetic factors on human performance is well documented, they are rarely addressed in interconnected, physiologically motivated hypotheses. This is especially true for multiple gene polymorphisms affecting interconnected metabolic systems compared to simple model systems [91,92,93]. Single gene polymorphisms rarely solely account for physiological variables [24], and especially VO_2_max [22,94]. 

Our investigation focused on exploring whether eleven selected gene polymorphisms affect the systemic cascade of oxygen transport during a cardiopulmonary exercise test. We assessed the evidence for a genetic influence using a Bayesian type of approach that compares the alternative hypothesis model of an effect with the null hypothesis of no effect based on the Bayes factor, BF10. Due to the complexity of possible hypotheses (see Figure 1), we used for practical reasons a Bayesian ANOVA with flat priors for hypothesis testing. Using this probabilistic statistical approach, we identified varied influences of certain polymorphisms on pulmonary, cardiovascular, and muscle metabolic oxygen transport variables. We found 24 moderately probable (BF10 < 10) and 12 strongly probable effects (BF10 > 10). The results identify novel genotype–phenotype associations, which seem plausible based on physiological principles rooted in molecular–biological and bioinformatic knowledge on the studied gene polymorphism [95,96]. Prior knowledge suggests these findings are unlikely to represent genetic confounding [97]. 

The observations (see Table 4) identify that multiple of the serially connected steps of oxygen transport are subject to a genetic influence and would have to be considered collectively to explain the inter-individual variability of (maximal) oxygen uptake [16]. Importantly, genetic influences also materialized at lower (metabolic) intensities of exercise [98,99,100]. The identified leverage points for genetic influences include multiple aspects that are described in terms of their resistive contribution to maximal oxygen uptake, i.e., cardiovascular blood transport to peripheral muscle tissue, including cardiac output and capillary-mediated oxygen transfer, and oxygen consumption in mitochondria, which provide energy equivalents for the fueling of muscle contraction [10,101]. 

**Figure 10:**
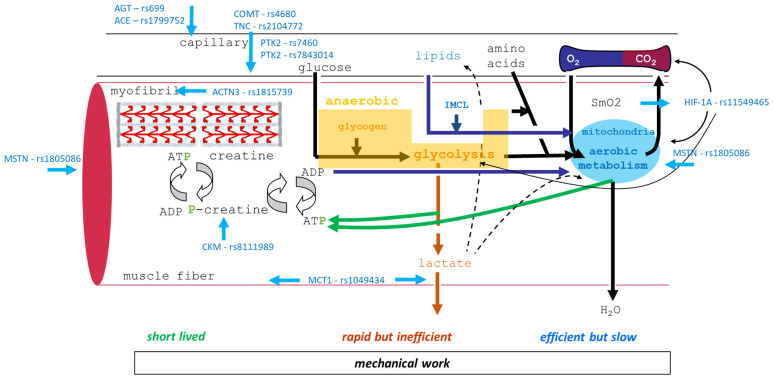
Sites of interaction of assessed gene polymorphism with metabolic and contractile processes involved in energy provision and force development by muscle fibers. Arrows point to demonstrated points of influence of anatomical/biochemical processes by the connected gene polymorphisms. Drawing of the cellular makeup of skeletal muscle in muscle fibers and capillaries and the embedded myofibrillar and mitochondrial organelles, as well as biochemical processes for fueling energetic requirements during physical work by means of the aerobic combustion of organic substrates. Arrows indicate sites of demonstrated influence of the eleven studied gene polymorphisms. We refer to the other illustrations and tables regarding the abbreviations.

Five key polymorphisms, rs1049434, rs11549465, rs4680, rs2104772, and rs7843014, in the genes for MCT1, HIF1A, COMT, TNC, and PTK2, were identified as influential for aerobic metabolism and performance. Integrating these genetic influences with existing knowledge explains their impact on the oxygen transport cascade, revealing both expected and unexpected effects on gene expression and aerobic metabolism. The biochemical and cellular influences are discussed and illustrated in Figure 10. 

The most extreme effect concerned the influence of polymorphism rs1049434 in the gene for the lactate transporter mono carboxylate transporter 1, MCT1, on SmO_2_ in the knee extensor muscle, *m. vastus lateralis*, and the ankle extensor muscle, *m. gastrocnemius*, at the start before graded exercise. Rs1049434 involves a glutamate-to-aspartate exchange at amino acid 490, affecting lactate transport in muscle fibers and erythrocytes [102,103]. It is associated with variability in aerobic metabolism and slow muscle fiber distribution, impacting lactate accumulation (reviewed in [103]). This lactate buildup indicates high strain on muscle metabolism, linked to mitochondrial activity and oxygen release from hemoglobin and myoglobin [104]. Lower SmO_2_ values in AA genotypes with an expected improved lactate transport compared to T-allele carriers suggest that the strength-based tests before the cardiopulmonary exercise test influenced aerobic muscle metabolism via lactate-related processes. Moderate evidence also shows rs1049434’s impact on tHb concentration in *m. vastus lateralis*, supporting the role of blood lactate in control of muscle perfusion via vasodilatation [105].

Another strongly probable genetic effect could be observed as an association between polymorphism rs11549465 in the HIF1A gene and RER at VT2. Gene polymorphism rs11549465 dictates a non-synonymous exchange of proline for serine at amino acid position 582 of the hypoxia-inducible transcription factor HIF1A in consequence of the substitution of C for T. This consequently reduces the stability of HIF1A as this protein only accrues under the physiological situation of tissue hypoxia with exercise (reviewed in [106,107,108]. This is expected to affect HIF1A-mediated control of the expression of batteries of proteins being associated with aerobic muscle metabolism [109,110], such as those enzymes involved in hemoglobin and capillary-mediated oxygen transport vis-a-vis mitochondrial respiration factors and glycolytic enzymes [109]. The identified influence of rs11549465 on RER at VT2 possibly reflects the reported association of this gene polymorphism with the ratio of glycolytic vs. mitochondria-associated enzymatic activities in skeletal muscles [110]. 

Polymorphism rs4680 in the gene for the catechol-O-methyltransferase enzyme, COMT, presented another example of a genetic influence on aerobic metabolism in skeletal muscle. Associations were found with SmO_2_ in vastus lateralis muscle at the start and VT1, and with hemoglobin concentration at VT2 and VO_2_max during recovery. Rs4680 affects blood-mediated oxygen metabolism in the forebrain during intense cognitive demand [35]. It involves a non-synonymous methionine-to-valine substitution at amino acid position 158, increasing COMT enzyme activity and the metabolism of dopamine, which is a classical regulator of vascular tone (reviewed in [37,42]). This consequently lowers the degree of dopamine-mediated influences on the contractility of smooth muscle, i.e., the constriction of blood vessels in C-allele carriers [36], which is expected to enhance perfusion of skeletal muscle with the onset of exercise. For CC genotypes compared to T-allele carriers, an elevated muscle tHb concentration is expected, reflecting the perfused vascular bed in peripheral tissues. This was observed at VT2 and VO_2_max during running exercise between CC and CT genotypes of rs4680. This was paralleled by a moderate probability for lower values in SmO_2_ in *vastus lateralis* muscle at the start of running in CC genotypes, which is indicative of the stimulation of aerobic metabolism in mitochondria. The implications of the potential dopaminergic mechanism for aerobic metabolism, the perfusion on contractile function, and the reported higher stress resilience of CC genotypes of rs4680 demand further investigation (reviewed in [37]). The previously reported association of COMT on suicidal ideation [77], as well as the predictive power of the serotonin transporter gene 5HTTLPR [78], further motivate future investigations in the tactical population.

Polymorphism rs2104772 in the tenascin-C gene strongly influenced mass-related maximal oxygen uptake and oxygen uptake at VT2, with AA genotypes showing higher VO_2_max values than TT genotypes (Figure 3). It also moderately affected tHb concentration in the vastus lateralis muscle. Tenascin-C impacts alveolar airway surface area and capillary growth, crucial for oxygen uptake [32], by setting the anatomical limits for pulmonary oxygen extraction [12], and it controls the growth of capillary structures [38,39,40]. We reported that gene polymorphism rs2104772 is associated with the variability in mitochondrial volume density and endurance-exercise-induced angiogenesis in *m. vastus lateralis* [34] as well as maximal oxygen uptake in skiing athletes [86]. This suggests that the further observed moderately probable influence of rs2104772 on power output at VT2 may relate to tenascin-C-related differences in pulmonary and cardiovascular elements of aerobic metabolism. The degree to which the aforementioned anatomical aspects explain this relationship remains to be further assessed. Likely, these associations relate to the altered molecular functioning of tenascin-C as a consequence of the non-synonymous exchange of leucine by isoleucine by the A-to-T substitution in amino acid codon 1677 of tenascin-C that is described by rs2104772 [34,111]. Collectively, the observations highlight a possibly important genetic contribution of tenascin-C to ventilatory aspects of the cascade of oxygen transport.

Intriguingly, highly probable genetic influences on the oxygen transport cascade were also identified for the polymorphism in the gene for protein tyrosine kinase 2, PTK2. PTK2 is involved in mechano-dependent signaling/proliferative responses that originate at sites of cell adhesion in part via a tenascin-C-related process [47,48]. This is important for the growth and development of tissues exposed to mechanical impacts, such as skeletal, smooth, and cardiac muscle, and endothelial cells lining the lumen of the vessels and lung [49,50,112,113]. In line with this prior knowledge, rs7843014 was strongly associated with specific oxygen uptake at low metabolic intensity (i.e., VT1) in the present study, while polymorphism rs2104772 in the functionally related tenascin-C gene was contrastingly associated with the specific oxygen uptake at VT2 and VO_2_max. This relates to the reported association of this gene polymorphism with peak oxygen uptake in skiing athletes [86]. Conversely, polymorphism rs7460 in the PTK2 gene was associated with cardiac output during running exercise and muscle concentration of tHb. The mechanistic bases of this observation, i.e., whether this possibly relates to the kinetics of the activation of PTK2 and tenascin-C signaling and/or consequent differences in PTK2 and tenascin-C regulated structures of oxygen transport, such as the regulation of muscle perfusion in contracting skeletal muscle [114], may contribute to explaining our findings. The present, novel observations, therefore, motivate further exploration of the interplay of the addressed polymorphism for the molecularly coupled related TNC and PTK genes for pulmonary and cardiovascular functioning.

We found evidence for the probable influence of rs7843014 on RER at VO_2_max. This may relate to the documented association of hypoxia-related signaling in vascular smooth muscle cells that control tissue perfusion and pulmonary gas exchange with the HIF1A-modulated posttranslational activation of PTK2 [115]. Support for a mechanistic convergence is provided by the post hoc observation that rs11549465 and rs7843014 gene polymorphism exert an extreme interaction effect on RER during loaded and graded exercise (BF10 > 100). This information provides input for attempts to individualize the (an)aerobic training response respective to the influence of exercise-induced tissue hypoxia and ambient hypoxia [106].

Interestingly, both polymorphisms, rs7843014 and rs7460, in protein tyrosine kinase 2 (also focal adhesion kinase), and to an anecdotal extent, rs2104772 in the functionally connected tenascin-C gene, were associated with body mass. This observation aligns with our initial evidence showing a strong probability of rs7843014 influencing body mass [116]. It also explains why the influence of rs7843014 on oxygen uptake at VT1 was anecdotal only when values were not standardized to body mass, with BF10 values of 0.835 versus 67.930. This suggests that part of the association of these gene polymorphisms with body mass-related (i.e., specific) oxygen uptake at different ventilatory thresholds may be due to the influence of body mass on aerobic energy expenditure during running exercise, thus warranting further investigation into the underlying mechanisms.

A notable observation was that the probability of the assessed gene polymorphisms affecting power output was only moderate, compared to the strongly probable influences on aspects of aerobic metabolism. This underscores the metabolic processes’ significant and likely less redundant role in determining endurance performance. In turn, this highlights that endurance performance is influenced by additional contractile and neuromuscular factors, of which only one directly involved gene, ACTN3, was studied.

Among the 24 moderately probable genetic influences, polymorphism rs699 in the AGT gene, like rs1799752 in the ACE gene, is linked to cardiovascular adjustments such as angiogenesis, mitochondrial metabolism, and muscle growth [28,117,118]. The absence of effects based on flat priors in the Bayesian statistics, except for a moderate influence of rs699 on aerobic power reserve, is surprising. This may be due to strength exercises performed before running that may override rs699- and rs1799752-associated differences in vasoconstriction [119]. As well, we note that additive effects of the genotypes alone were assessed, but when over-dominant effects have been reported to occur for perfusion-associated fatigue resistance for rs1799752 [114]. Cardiovascular parameters are influenced by rs1799752 in interaction with rs699 [120]. Indeed, we identified that rs1799752 exerted an influence on the hemoglobin concentration in knee extensor muscle during recovery from running into exhaustion, in line with our previous report [121]. Despite strong indications of the renin–angiotensin system’s role in perfusion-related processes that support aerobic performance [119,122,123], the genetic mechanisms in tactical athletes require further investigation. 

Additionally, there is evidence for a moderate probability of influence of rs1815739 on oxygen saturation of the vastus lateralis muscle at VO_2_max, cardiac output, and anaerobic power reserve. The ACTN3 gene, associated with rs1815739, encodes a cytoskeletal element that stabilizes fast-twitch muscle fibers, affecting force production and blood pressure during exercise stress testing in elite male athletes, possibly through peripheral blood vessel constriction [124]. T-allele carriers of rs1815739 are expected to have lower percentages of fast-twitch muscle fibers, which produce higher forces during contraction but with reduced efficiency (reviewed in [61,125]). The lack of influence of rs1815739 on tHb concentration in vastus lateralis muscle suggests that occluded blood flow is not the cause of the moderate influence on SmO_2_ at VO_2_max. Instead, differences in mitochondrial activity rates between rs1815739 genotypes indicate variations in metabolic efficiency due to fiber type composition [61]. This contention is supported by the moderate influence of rs1815739 on the anaerobic power reserve, aligning with the expected higher values in the more efficient muscles of rs1815739 T-allele carriers (Figure 9), who may have a higher percentage of slow-twitch muscle fibers [21,61]. 

Finally, we note a moderate probability of rs11549465-associated differences in power output at VT2 and VO_2_max, which relates to the observed strong influence on RER at VT2, whereby higher values were revealed in CC than TT genotypes for body mass-related power output and RER (compare Figure 6 with Figure 9). This corroborates the contention of a positive impact of carrying the C-allele for the manifestation of aerobic capacity [94].

Few genotype effects align with our prior knowledge on genotype–phenotype associations (compare Figure 1 with Table 4). This concerns, for instance, the influence of rs2104772 on specific oxygen uptake [86] and the influence of rs11549465 on RER, consistent with the role of the encoded transcription factor HIF1A in regulating mitochondrial respiration [126]. Similarly, the probable influence of rs4680 on SmO_2_ and tHb concentration in the *vastus lateralis* muscle during strenuous running aligns with the reported influence of rs4680 on perfusion-related aerobic metabolism in the forebrain during stressful cognitive [35]. 

Interestingly, our investigation did not confirm previously identified associations of rs11549465 with maximal aerobic capacity or performance [94,108], nor rs1799752 with muscle oxygen saturation during dynamic exercise to fatigue [29]. For the polymorphism rs1805086 in the MSTN gene, an alternative hypothesis may explain this observation. For example, power output was higher in TT- than TC-rs1805086 genotypes at VT1 and VO_2_max during endurance exercise, whereas meta-data would predict lower power for TT vs. TC genotypes during short single contractions [64,65]. The T-to-C change in rs1805086 results in a non-synonymous substitution of lysine for arginine at amino acid position 153, potentially unfavorable for MSTN’s molecular binding. 

Our observations align with an inhibitory influence of the C-allele of rs1805086 on MSTN function, which has been found to reduce leg muscle fatigue resistance and aerobic capacity in MSTN-deficient versus wildtype mice [63]. Our findings may reflect an inverse relationship between maximal power for short contractions and repeated fatiguing contractions, due to myofibril and mitochondrial contributions. We propose that TT genotypes show better muscle power during endurance exercise, while the opposite may be true for maximal power during short contractions. Our findings may reflect the inverse relationship between maximal power values for short contractions and repeated fatiguing contractions due to reciprocal myofibril and mitochondria contributions to the volume density and muscle fiber types in power and endurance-trained subjects [1,127]. Thus, we propose a complementary hypothesis where TT genotypes vs. heterozygous C-allele carriers of rs1805086 demonstrate better muscle power during endurance exercise. Nevertheless, the opposite may be true for maximal power during short single contractions.

A notable observation was that the distribution of six gene polymorphisms in the studied population of tactical athletes deviated from the Hardy–Weinberg equilibrium. To the best of our knowledge, this is the first investigation of the association of endurance performance in a cohort of tactical Swiss athletes. Several factors, including methodological aspects and conditions that are not compliant with the assumption for an equilibrium of transmitted alleles, may explain this observation [128,129]. We formally exclude the possibility of major technical flaws, as the deployed methods were established and validated previously [86]. Care was taken to limit inferences from inappropriate genotyping by sequencing samples where the confidence in genotype estimation by high-resolution melting curve was low. A departure from the Hardy–Weinberg equilibrium has been reported before for the conspicuous polymorphisms, i.e., rs4680, in a control study with Caucasians, and it was concluded that this may reflect a potential limitation in population stratification rather than the quality of genotyping [130]. The volunteering subjects in our investigation were likely self-selected based on their interest in qualifying as future operators. This may have increased the statistical significance of the reported enriched presence of certain alleles for the polymorphisms rs1799752, rs4680, rs1049434, and rs8111989 in subjects with endurance or strength phenotypes of physical fitness [26,32,33,34,35,36,37,38,39,40,47,48,49,50,57]. The non-equilibrated distribution of polymorphisms in the respective genes also reflects the association of the encoded gene products (i.e., ACE, COMT, MCT1, and possibly CKM) with aerobic metabolism in mitochondria [30,35,103,131], which are not inherited in a Mendelian fashion [132], thus violating some assumptions of the Hardy–Weinberg equilibrium. Indeed, it has been previously reported that the ACTN3 gene polymorphism rs1815739 also deviates from Hardy–Weinberg equilibrium in athletes of the Swiss national skiing team [86]. Future investigations may need to address the potential for constrained selection in genetic studies with tactical athletes from other geographic regions.

Limitations: Several factors warrant discussion regarding their influence on the resolved effects and interpretations. Firstly, due to the complexity and rarity of minor alleles for some impactful genotypes, we were unable to systematically assess hypothesized interactions between multiple studied gene polymorphisms and, therefore, refrained from pursuing this analysis. While we applied methodological measures to ensure the quality of data collection from subjects as they underwent the battery of tests and constrained the study population to a comparable ethnic and habitual milieu, we still noted confounding influences.

Although the MetaMax 3B-R2 system is considered one of the most precise systems on the market, the percentage error (%e) for the MetaMax for the VO_2_ parameter is 1.64% and for the overall system it is 1.95% [133]. Diurnal variation in terms of mean difference in VO_2_max is 5.0 ± 2.0 mL/min/kg [134]. Due to the limited availability of the participants, we could not standardize the measurement times. No test took place before 10 a.m. or after 18 p.m.; however, we cannot rule out differences in response variables due to different CPET times of the day and time of the year. 

For instance, we identified a few outliers in the near-infrared-based measurements for SmO_2_ and tHb, which were removed from the final statistical analysis. Additionally, the physiological state in which the subjects performed the loaded-ramped running was likely influenced by the assessment of their physical and psychological performance as tactical athletes. This may have masked or revealed possible genotype–phenotype associations. For example, intense strength exercises prior to the loaded ramped running likely affected the perfusion of peripheral tissues, potentially interfering with the detection of the hypothesized influence of rs1799752 on oxygen saturation and tHb concentration in *m. vastus lateralis* and *m. gastrocnemius* during running, as the involved angiotensin 2 system depends on exercise-induced perfusion [119]. Similarly, we find support for the view that prior exercise influenced the hypothesized effects of the gene polymorphism rs1049434, affecting the transport of lactate that accumulates during strenuous exercise (reviewed in [135]). This compound has been postulated to influence aspects of oxygen transport in peripheral muscle tissue, including the release of bound oxygen from hemoglobin in capillaries and myoglobin deoxygenation, detectable via SmO_2_ and tHb [104]. Interestingly, we noted very strong probable influences of rs1049434 on SmO_2_ in *m. vastus lateralis* and *m. gastrocnemius* during the subsequent graded protocol of running to exhaustion. This contrasts with indications of enhanced lactate accumulation towards the end of the graded running test, based on high RER values (i.e., 1.38), which imply a substantial contribution of anaerobic glucose metabolism to meet the metabolic requirements of the contracting peripheral muscle. The role of MCT1-mediated lactate efflux kinetics vis-à-vis lactate accumulation for SmO_2_ in the studied knee and ankle extensor groups, and the role played by rs1049434, requires further investigation [136]. 

Finally, we identify that few female athletes were included in this study, although not in sufficient number to allow a generalization across sexes.

## 5. Conclusions

Our observations reveal varied influences of sequence variants in genes that determine the expression of aerobic metabolic traits during intense muscle work on pulmonary, cardiovascular, and muscle metabolic oxygen transport variables in tactical athletes. The findings can be interpreted based on a prior mechanistic understanding of the molecular and cellular influence of the selected gene polymorphism regarding leverage points (such as anatomical differences in capillary density, mitochondrial volume density, and fiber type composition) at which the oxygen transport cascade might be subject to a functionally beneficial genetic influence. 

In our sample of 251 well-trained tactical athletes, a few relevant genetic influences on the oxygen transport cascade were identified, affecting skeletal muscle aerobic metabolism. Key genotype–phenotype relationships include MCT1 (rs1049434) and COMT (rs4680) with SmO_2_ and tHb in knee extensor muscle, HIF1A (rs11549465) and PTK2 (rs7843014) with RER, PTK2 (rs7843014) with body mass, and PTK2 (rs7460) with cardiac output. Other associations, like TNC (rs2104772) with maximal oxygen uptake, were confirmed [86]. 

These findings suggest that along with physiological factors, exercise, and nutrition, genetic factors significantly influence aerobic capacity. Future research may develop prescriptive recommendations for specific training stimuli to enhance endurance performance based on the knowledge of the individual genetic profile. Notably, COMT (rs4680) affects both skeletal muscle metabolism during exercise and forebrain function during cognitive tasks, indicating associations with stress resilience or even post-traumatic stress disorder. The absence of influences of angiotensin-related polymorphisms (rs699 and rs1799752) on aerobic performance warrants further investigation, considering potential confounding factors.

## Figures and Tables

**Table 1:** Summary of deployed PCR conditions. Key information used to detect the gene polymorphisms under investigation based on real-time PCR on genomic DNA. Deployed protocols comprised a generic denaturation step at 2 min 95 °C, followed by the in-here-named specific conditions of a thermal cycling protocol that was followed by a final extension step for 120 s at 72 °C before a ramp protocol was run to determine the respective polymorphism for the amplified DNA product based on a specific melting curve.

Gene	Nucleotide	Rside	Oligonucleotide (Sense)	Oligonucleotide (Anti-Sense)	Cycling Conditions	Ramp Protocol	Reagent
AGT #	(frame)	rs699	CCATCTCCAAGGCCTGACTG	TATACCCCTGTGGTCCTCCC	35 × [15″ @ 95 °C, 30′ @ 55 °C, 30″ @ 72 °C]	55–95 °C, 0.3 °C s^−1^	KAPA SYBR Fast
	T or C	rs699	TAGGTGTTGAAAGCCAGGGTG	GTGACAGGATGGAAGACTGGC	40 × [15″ @ 95 °C, 60″ @ 55 °C]	50–95 °C, 0.1 °C s^−1^	SensiFAST
ACE	I	rs1799752	TGGGATTACAGGCGTGATACAG	AATTTCAGAGCTGGAATAAAATT	40 × [15″ @ 95 °C, 60″ @ 55 °C]	60–95 °C, 0.3 °C s^−1^	KAPA SYBR Fast
	D	rs1799752	CATCCTTTCTCCCATTTCTC	AATTTCAGAGCTGGAATAAAATT	40 × [15″ @ 95 °C, 60″ @ 55 °C]	60–95 °C, 0.3 °C s^−1^	KAPA SYBR Fast
COMT	T	rs4680	CGCGGCCGGCCGATGGTGGATTTCGCTGGAA	TTTCCAGGTCTGACAACGG	45 × [15″ @ 95 °C, 60′ @ 63 °C]	60–95 °C, 0.05 °C s^−1^	KAPA SYBR Fast
	C	rs4680	GATGGTGGATTTCGCTGGAG	TTTCCAGGTCTGACAACGG	45 × [15″ @ 95 °C, 60′ @ 63 °C]	60–95 °C, 0.05 °C s^−1^	KAPA SYBR Fast
TNC	A or T	rs2104772	CAAAAAAAGCAGTCTCTGAGCCAC	TTCAGTAGTCTCTCTCTGAGAC	40 × [15″ @ 95 °C, 15″ @ 60 °C, 30″ @ 72 °C]	55–95 °C, 0.1 °C s^−1^	SensiFAST
PTK2	T or A	rs7460	TGGGTCGGGAACTAGCTGTA	ATGGAAAAAGGGGATGGTCC	40 × [15″ @ 95 °C, 15″ @ 60 °C, 30″ @ 72 °C]	55–95 °C, 0.1 °C s^−1^	SensiFAST HRM kit
PTK2	C or A	rs7843014	TGATGGGACCTAAACCCATT	TTTCCCATCAGCTGCTTGTT	40 × [15″ @ 95 °C, 15″ @ 60 °C, 30 @ 72 °C]	55–95 °C, 0.1 °C s^−1^	SensiFAST HRM kit
HIF-1A	C or T	rs11549465	CCTCCAGTTACGTTCCTTCG	TGAGGACTTGCGCTTTCAGG	40 × [15″ @ 95 °C, 15″ @ 55 °C, 30″ @ 72 °C]	55–95 °C, 0.1 °C s^−1^	SensiFAST
MCT1	A or T	rs1049434	AGATGTTGCTGGGAAGCCAA	CTTCAGCCCCATGGATTCAG	40 × [15″ @ 95 °C, 15″ @ 55 °C, 30″ @ 72 °C]	50–95 °C, 0.1 °C s^−1^	SensiFAST
CKM	T or C	rs8111989	TTCAGTGTGGCCTTGAGTTG	GCTGCCAGTCCCTTACTTTC	40 × [15″ @ 95 °C, 15″ @ 55 °C, 30″ @ 72 °C]	55–95 °C, 0.1 °C s^−1^	SensiFAST HRM kit
ACTN3	T or C	rs1815739	ACACTTCCTGCCTGTCGTCC	GATCTTCTGGATCTCACCCTGG	40 × [15″ @ 95 °C, 15″ @ 60 °C, 30″ @ 72 °C]	65–95 °C, 0.3 °C s^−1^	SensiFAST
MSTN	T or C	rs1805086	TGGAAAACCCAAATGTTGCTTC	AGTCTCGACGGGTCTCAAAT	40 × [15″ @ 95 °C, 15″ @ 50 °C, 30″ @ 72 °C]	55–95 °C, 0.1 °C s^−1^	SensiFAST HRM kit

Abbreviations: HRM, high resolution melt; KAPA SYBR FAST, KAPA SYBR FAST Mix (ABI Prism, Merck); SensiFAST, SensiFAST HRM Mix (Meridian Biosciences, Labgene); #, nested approach with 1:40,000 dilution of a framing PCR as a first step.

**Table 2:** Physiological characteristics. Mean ± SE [minima–maxima] of anthropometric and functional parameters of the 251 assessed subjects before and during loaded ramped running exercise of the assessed subjects.

Parameter	Unit	Mean ± SE	[Min–Max]
age	[years]	28.16 ± 0.46	[18.00–52.00]
body mass	[kg]	79.59 ± 0.69	[52.25–111.40]
body height	[cm]	179.40 ± 0.45	[161.50–201.00]
BMI	[kg m^−2^]	24.68 ± 0.17	[18.40–34.60]
strength training	[%]	45.38 ± 1.69	[0.00–100.00]
endurance training	[%]	35.20 ± 1.49	[0.00–100.00]
tactical and other training	[%]	13.50 ± 1.14	[0.00–100.00]
VO_2_max	[L O_2_ min^−1^]	4.26 ± 0.04	[2.29–6.28]
max cardiac output	[L min^−1^]	26.00 ± 0.25	[13.99–37.47]
max heart rate	[beats min^−1^]	188.34 ± 0.71	[106.00–212.00]
SmO_2_ VAS VO_2_max	[%]	14.93 ± 0.78	[0.50–77.50]
SmO_2_ GAS VO_2_max	[%]	17.50 ± 0.66	[1.00–41.90]
tHb VAS VO_2_max	[%]	12.59 ± 0.03	[11.43–13.43]
time to exhaustion	[sec]	556.6 ± 6.5	[300–730]
max RER	[mL CO_2_/mL O_2_]	1.38 ± 0.01	[0.99–1.70]
max lactate	[mM]	13.69 ± 0.21	[5.44–23.30]
max aerobic power	[W kg^−1^]	3.64 ± 0.05	[1.70–5.31]
anaerobic power reserve	[Watt kg^−1^]	0.88 ± 0.03	[0.11–3.01]

Abbreviations: BMI, body mass index; max, maximal; n, number of evaluated data points; RER, respiration exchange ratio; SE, standard error; VO_2_max, maximal oxygen uptake.

**Table 3:** Distribution of genotypes and *p*-values respective to the Hardy–Weinberg equilibrium as assessed with Chi2-tests for the 251 assessed subjects. M and m denote major and minor variants of the nucleotide for the respective gene polymorphism. ACE, COMT, PTK2, HIF1A, MCT1, and CKM show deviation from the equilibrium.

Gene	Rsid	M/m	MM	Mm	mm	*p*-Value
AGT	rs699	T/C	29.08%	54.98%	15.94%	0.0600
ACE	rs1799752	I/D	17.53%	63.75%	18.73%	<0.001
COMT	rs4680	T/C	35.38%	31.28%	33.33%	<0.001
TNC	rs2104772	A/T	26.69%	51.79%	21.51%	0.540
PTK2	rs7460	T/A	14.34%	45.82%	39.84%	0.752
PTK2	rs7843014	C/A	20.72%	39.84%	39.44%	0.006
HIF1A	rs11549465	C/T	76.89%	7.17%	15.94%	<0.001
MCT1	rs1049434	A/T	49.40%	36.25%	14.34%	0.006
CKM	rs8111989	T/C	60.16%	31.47%	8.37%	0.027
ACTN3	rs1815739	T/C	3.98%	36.65%	59.36%	0.364
MSTN	rs1805086	T/C	94.42%	5.58%	0.00%	0.650

**Table 4:** Summary of genetic effects on response variables during aerobic muscle work. Bayes factors (BF10) for effects on power output and selected metabolic parameters during the test of loaded ramped exercise to exhaustion. Bayesian repeated-measures ANOVA or univariate Bayes ANOVA was used. Observations favoring the alternative hypothesis compared to the null hypothesis on a relevant genetic influence (based on a rounded BF10 of 3) are highlighted in a gray cell. Dark gray highlighted values correspond to those being of main relevance for the hypotheses. BF10 values representing a strong probability of an effect are highlighted in bold. The values for the parameters ‘overall GP’, ‘RM’, and ‘RM + GP’ refer to those from the repeated measures ANOVA. Those values denoted as ‘max GP’, ‘start’, ‘VT1’, ‘VT2’, and ‘VO_2_max’ correspond to the BF10 values for the post hoc test, whereby ‘max GP’ refers to the highest value for the post hoc effects of a genotype.

	Polymorphism	rs699	rs1799752	rs4680	rs2104772	rs7460	rs7843014	rs11549465	rs1049434	rs8111989	rs1815739	rs1805086
	Process	CVASC	CVASC	CVAS	CVASC	CVASC	CVASC	Aemet and Gly	Aemet	Charging F	Develop F	Maintain F
Parameter	Time Point/Gene	AGT	ACE	COMT	TNC	PTK2	PTK2	HIF1A	MCT1	CKM	ACTN3	MSTN
oxygen uptake [mL O_2_ min^−1^ kg^−1^]	overall GP	0.052	0.035	0.032	0.378	0.027	0.378	0.083	0.028	0.071	0.06	0.217
	max GP	0.280	0.157	0.171	2.768	0.139	1.818	0.298	0.151	0.361	0.391	0.217
	RM	5.72 × 10 + 230	5.72 × 10 + 230	5.72 × 10 + 230	5.72 × 10 + 230	5.72 × 10 + 230	5.72 × 10 + 230	5.72 × 10 + 230	5.72 × 10 + 230	5.72 × 10 + 230	5.72 × 10 + 230	5.67 × 10 + 230
	RM + GP	1.58 × 10 + 230	1.09 × 10 + 230	1.04 × 10 + 230	1.24 × 10 + 231	8.54 × 10 + 229	1.38 × 10 + 231	2.03 × 10 + 230	8.04 × 10 + 229	1.97 × 10 + 230	1.34 × 10 + 230	3.24 × 10 + 230
	VT1	0.380	0.372	0.581	0.392	1.116	67.930	0.289	0.484	0.408	0.803	0.463
	VT2	0.515	0.225	0.240	138.262	0.234	0.904	0.687	0.218	0.790	0.380	0.308
	VO_2_max	0.450	0.267	0.204	5.934	0.212	1.443	0.415	0.243	0.373	0.642	0.297
RER	overall GP	0.026	0.034	0.02	0.061	0.025	0.123	0.196	0.026	0.035	0.04	0.309
	max GP	0.155	0.171	0.111	0.302	0.136	0.648	0.547	0.135	0.172	0.233	0.216
	RM	1.693 × 10 + 251	1.674 × 10 + 251	1.674 × 10 + 251	1.674 × 10 + 251	1.674 × 10 + 251	1.674 × 10 + 251	1.693 × 10 + 251	1.693 × 10 + 251	1.693 × 10 + 251	1.693 × 10 + 251	1.693 × 10 + 251
	RM + GP	1.630 × 10 + 250	2.480 × 10 + 250	1.059 × 10 + 250	1.042 × 10 + 251	1.559 × 10 + 250	5.523 × 10 + 251	7.423 × 10 + 251	1.396 × 10 + 250	2.347 × 10 + 250	2.007 × 10 + 250	6.893 × 10 + 250
	VT1	0.215	1.248	0.335	0.871	0.525	0.743	2.041	0.311	0.428	1.941	0.464
	VT2	0.300	0.332	0.288	0.675	0.393	3.37	19.971	0.231	0.313	0.341	0.314
	VO_2_max	1.335	0.263	0.287	2.931	0.307	12.750	1.401	0.219	0.256	0.498	0.318
cardiac output [L min^−1^]	overall GP	0.188	0.106	0.088	0.134	0.729	0.193	0.21	0.183	0.347	0.49	0.399
	max GP	0.611	0.171	0.113	0.306	22.036	0.601	0.292	0.775	3.425	2.694	0.399
	RM	1.24 × 10 + 193	1.24 × 10 + 193	1.27 × 10 + 193	1.39 × 10 + 193	2.72 × 10 + 193	1.27 × 10 + 193	1.27 × 10 + 193	1.27 × 10 + 193	1.23 × 10 + 193	1.27 × 10 + 193	1.23 × 10 + 193
	RM + GP	6.18 × 10 + 192	4.71 × 10 + 192	3.86 × 10 + 192	4.56 × 10 + 192	1.39 × 10 + 193	6.4 × 10 + 192	6.79 × 10 + 192	5.91 × 10 + 192	6.12 × 10 + 192	1.08 × 10 + 193	8.45 × 10 + 192
	VT1	0.377	0.289	0.184	0.255	2.422	0.661	0.286	0.643	0.679	0.395	0.370
	VT2	0.384	0.230	0.177	0.382	1.448	0.285	0.311	0.344	0.966	0.485	0.306
	VO_2_max	0.416	0.233	0.216	0.305	2.015	0.309	0.293	0.345	0.562	0.348	0.317
tHb *m. vastus lateralis* [g dL^−1^]	overall GP	0.46	0.559	2.415	0.499	0.387	0.43	0.517	0.797	0.479	0.48	0.683
	max GP	0.569	2.287	17.426	2.562	11.678	0.771	0.363	6.639	0.273	0.234	1.076
	RM	1.916 × 10 + 29	1.915 × 10 + 29	1.916 × 10 + 29	1.916 × 10 + 29	1.916 × 10 + 29	1.916 × 10 + 29	1.916 × 10 + 29	1.916 × 10 + 29	1.916 × 10 + 29	1.916 × 10 + 29	1.916 × 10 + 29
	RM + GP	1.105 × 10 + 29	1.071 × 10 + 29	3.976 × 10 + 29	1.059 × 10 + 29	2.653 × 10 + 29	9.463 × 10 + 28	1.016 × 10 + 29	1.097 × 10 + 29	9.837 × 10 + 28	1.017 × 10 + 29	1.376 × 10 + 29
	Start	0.243	0.490	0.674	0.291	1.449	0.221	0.696	2.411	0.407	0.417	0.553
	VT1	0.280	0.330	1.428	0.242	0.564	0.294	0.382	0.412	0.404	0.385	0.393
	VT2	0.336	0.456	9.211	0.475	0.593	0.424	0.341	0.448	0.298	0.398	0.779
	VO_2_max	0.284	0.801	2.561	0.275	0.593	0.291	0.339	0.950	0.285	0.389	0.552
Sm O_2_ *m. vastus lateralis* [%]	overall GP	0.110	0.129	0.206	0.084	0.051	0.106	0.075	0.187	0.073	0.371	0.525
	max GP	0.527	0.487	1.722	0.419	0.221	0.615	0.236	0.931	0.293	0.705	0.521
	RM	1.517 × 10 + 101	1.517 × 10 + 101	1.517 × 10 + 101	1.517 × 10 + 101	1.517 × 10 + 101	1.517 × 10 + 101	1.517 × 10 + 101	1.517 × 10 + 101	1.517 × 10 + 101	1.521 × 10 + 101	1.518 × 10 + 101
	RM + GP	4.611 × 10 + 100	5.325 × 10 + 100	1.054 × 10 + 101	3.386 × 10 + 100	2.311 × 10 + 100	4.496 × 10 + 100	2.731 × 10 + 100	8.242 × 10 + 100	2.958 × 10 + 100	1.177 × 10 + 101	1.111 × 10 + 101
	Start	0.394	1.37	2.934	1.467	0.419	0.801	0.374	1080.411	0.352	0.874	0.329
	VT1	0.264	0.805	4.442	0.321	0.313	1.05	0.347	54.682	0.705	0.459	1.148
	VT2	0.809	1.150	0.562	0.333	0.279	0.828	0.346	0.291	0.504	0.911	0.426
	VO_2_max	1.532	0.289	0.211	0.563	0.393	0.670	0.363	0.487	0.298	3.385	0.317
Sm O_2_ *m. gastrocnemius* [%]	overall GP	0.108	0.054	0.091	0.191	0.106	0.05	0.306	0.163	0.065	0.168	0.378
	max GP	0.277	0.160	0.299	0.700	0.477	0.146	1.395	0.712	0.218	0.483	0.367
	RM	3.896 × 10 + 44	3.896 × 10 + 44	3.896 × 10 + 44	3.896 × 10 + 44	9.072 × 10 + 46	9.072 × 10 + 46	9.072 × 10 + 46	9.072 × 10 + 46	9.072 × 10 + 46	9.105 × 10 + 46	9.165 × 10 + 46
	RM + GP	1.709 × 10 + 46	9.124 × 10 + 45	1.445 × 10 + 46	3.016 × 10 + 46	1.556 × 10 + 46	8.185 × 10 + 45	4.189 × 10 + 46	2.547 × 10 + 46	1.054 × 10 + 46	2.352 × 10 + 46	4.415 × 10 + 46
	Start	0.318	0.262	0.982	0.281	2.851	0.378	0.390	121.206	0.438	0.673	0.299
	VT1	0.264	0.359	0.689	0.313	0.601	0.332	7.511	0.643	0.303	0.567	0.302
	VT2	1.136	0.276	0.292	0.316	1.337	0.629	1.075	0.834	0.288	1.699	0.526
	VO_2_max	1.888	0.289	0.857	0.527	0.525	1.857	0.632	5.475	0.302	0.277	1.780
tHb recovery *m. vastus lateralis* [g dL^−1^]	overall GP	0.408	0.613	4.422	0.266	0.425	0.534	0.445	0.549	0.290	0.327	0.676
	max GP	0.396	1.785	137.344	0.198	1.519	1.285	0.59	0.967	0.254	0.352	0.391
	RM	0.160	363.206	1.900	0.160	0.160	0.160	0.160	0.160	0.160	0.152	0.158
	RM + GP	0.062	78.365	0.160	0.042	0.063	0.083	0.071	0.083	0.046	0.050	0.105
SmO_2_ recovery *m. vastus lateralis* [%]	overall GP	0.041	0.056	0.035	0.042	0.044	0.040	0.075	0.042	0.051	0.082	0.227
	max GP	0.183	0.218	0.148	0.000	0.196	0.184	0.264	0.186	0.218	0.388	0.221
	RM	9.113 × 10 + 176	9.113 × 10 + 176	9.113 × 10 + 176	1.258 × 10 + 177	9.113 × 10 + 176	9.113 × 10 + 176	9.113 × 10 + 176	9.113 × 10 + 176	9.113 × 10 + 176	9.113 × 10 + 176	9.230 × 10 + 176
	RM + GP	5.074 × 10 + 175	6.105 × 10 + 176	6.175 × 10 + 175	6.805 × 10 + 175	7.551 × 10 + 175	1.033 × 10 + 176	3.579 × 10 + 176	5.614 × 10 + 175	9.130 × 10 + 175	1.014 × 10 + 176	2.390 × 10 + 176
SmO_2_ GAS recovery [%]	overall GP	0.049	0.047	0.041	0.049	0.052	0.048	0.086	0.046	0.054	0.104	0.268
	max GP	0.194	0.197	0.156	0.200	0.212	0.186	0.277	0.194	0.221	0.479	0.257
	RM	1.279 × 10 + 168	1.279 × 10 + 168	1.279 × 10 + 168	1.279 × 10 + 168	1.279 × 10 + 168	1.279×10 + 168	1.279 × 10 + 168	1.279 × 10 + 168	1.279 × 10 + 168	1.290 × 10 + 168	1.265 × 10 + 168
	RM + GP	1.337 × 10 + 167	8.462 × 10 + 166	1.076 ×10 + 167	1.410 × 10 + 167	1.109 × 10 + 167	4.126×10 + 167	9.265 × 10 + 167	7.615 × 10 + 166	1.098 × 10 + 167	3.482 × 10 + 167	8.962 × 10 + 167
performance [W kg^−1^]	overall GP	0.028	0.028	0.049	0.036	0.026	0.042	0.108	0.026	0.035	0.049	0.211
	max GP	0.157	0.142	0.143	0.196	0.147	0.247	0.353	0.155	0.163	0.261	0.211
	RM	6.474 × 10 + 269	6.474 × 10 + 269	6.474 × 10 + 269	6.474 × 10 + 269	6.474 × 10 + 269	6.474 × 10 + 269	6.474 × 10 + 269	6.474 × 10 + 269	6.474 × 10 + 269	6.427 × 10 + 269	6.514 × 10 + 269
	RM + GP	8.057 × 10 + 268	6.499 × 10 + 268	1.023 × 10 + 269	1.406 × 10 + 269	6.553 × 10 + 268	2.694 × 10 + 269	7.487 × 10 + 269	7.416 × 10 + 268	1.043 × 10 + 269	1.454 × 10 + 269	2.7 × 10 + 269
	VT1	1.096	0.338	1.175	0.293	5.000	1.268	0.287	0.216	0.495	3.795	0.418
	VT2	0.322	0.284	0.319	4.003	0.266	0.377	4.694	0.310	0.323	0.334	0.323
	VO_2_max	0.547	0.280	0.232	0.769	0.263	0.943	2.884	0.477	0.260	0.410	0.362
anaerobic power reserve	overall GP	2.507	0.225	0.269	0.586	0.545	0.193	2.176	0.380	1.356	2.728	0.369
body mass [kg]	overall GP	0.101	0.064	0.072	0.465	1.045	7.005	0.085	0.073	0.075	0.211	0.286
	max GP	0.404	0.221	0.282	1.559	3.555	26.05	0.309	0.305	0.297	0.465	0.286

Abbreviations: aemet, aerobic metabolism; CVASC, cardiovascular system; F, force; gly, glycolytic metabolism; GP, gene polymorphism; RM, repeated factor of exercise intensity or time into or post-exercise; RM + GP, isolated effect of GP in function of exercise intensity.

## Data Availability

The data presented in this study are available upon request from the corresponding author due to restrictions related to the protected nature of the subjects studied.

## Supplementary Materials

The following supporting information can be downloaded at: https://www.mdpi.com/article/10.3390/genes15121535/s1. Figure S1: Descriptive assessment of the normal distribution of the data based on histograms. Figure S2: Descriptive assessment of the normal distribution of the data based on QQ plots. Figure S3: Genotype effects on recovery of oxygen saturation and total hemoglobin concentration of vastus lateralis muscle after exhaustive ramp-loaded running exercise. Table S1: Descriptive statistics of the assessed proxy variables of systemic oxygen transport.
