# Supplementary material for: Genotypic Influences on Actuators of Aerobic Performance in Tactical Athletes
Source: Genes (Basel). 2024 Nov 28;15(12):1535. doi: 10.3390/genes15121535 (PMC11675622; doi:10.3390/genes15121535)
Supplement: Supplementary file 1 [file genes-15-01535-s001.zip › Figure S1-S2-R1.pdf]

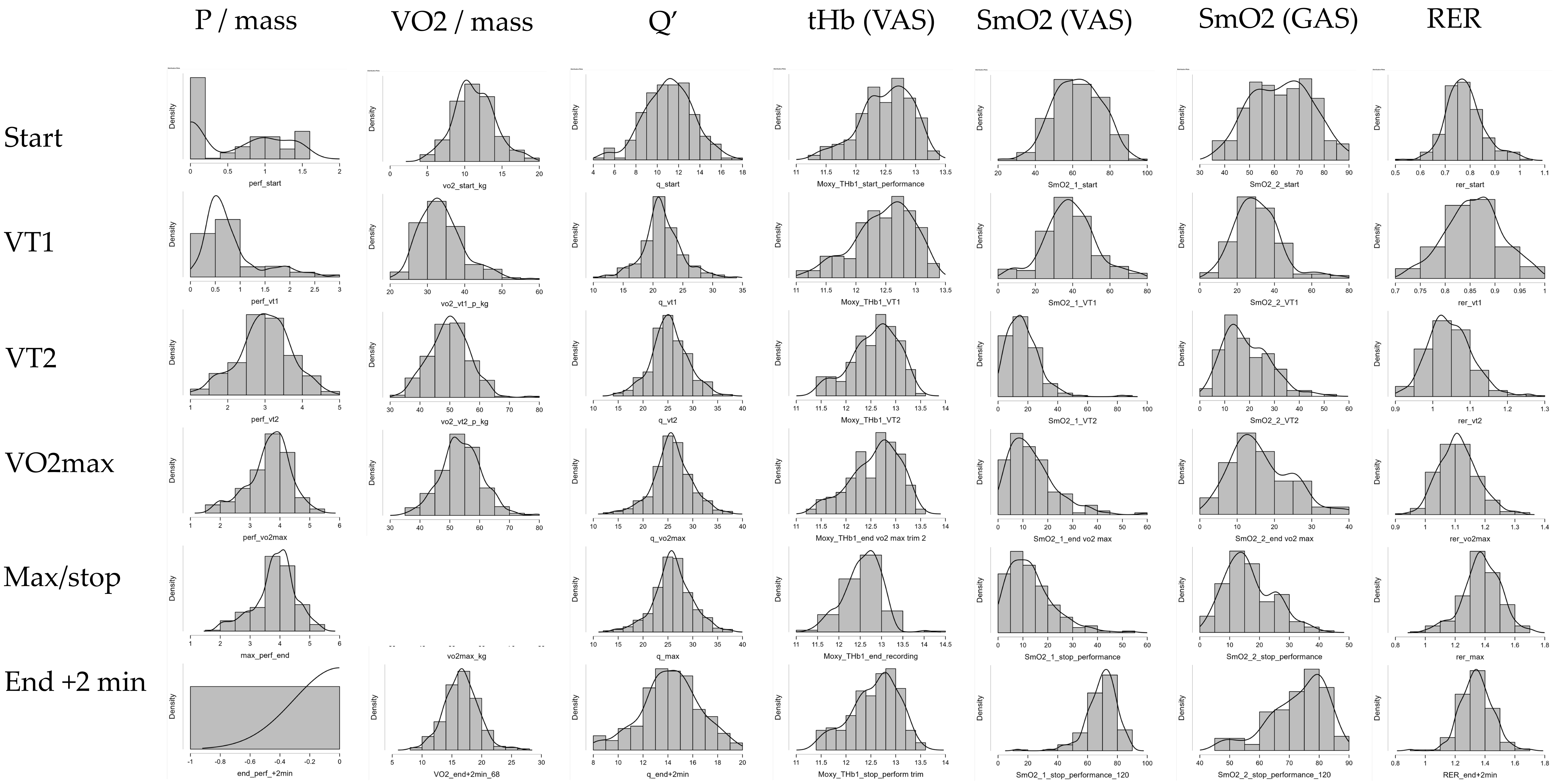

Figure S1. *Histograms of the distribution of assessed proxy variables of systemic oxygen transport.* Data were calculated from the avaiable data from the 251 studied subjects at the different intensities (start, VT1, VT2, VO2max,end + 2min). Individual panels at the same vertical height correspond to the intensity given to the far left of the figure. X-axes refer to the values for the respective proxy variable at a given intensity. Y-Axes refer to the density of the presence of data for the selected inverval of values in the x-axis. Abbreviations: VAS, m. vastus lateralis; GAS, m. gastrocnemius. Further information is given in the main manuscript.

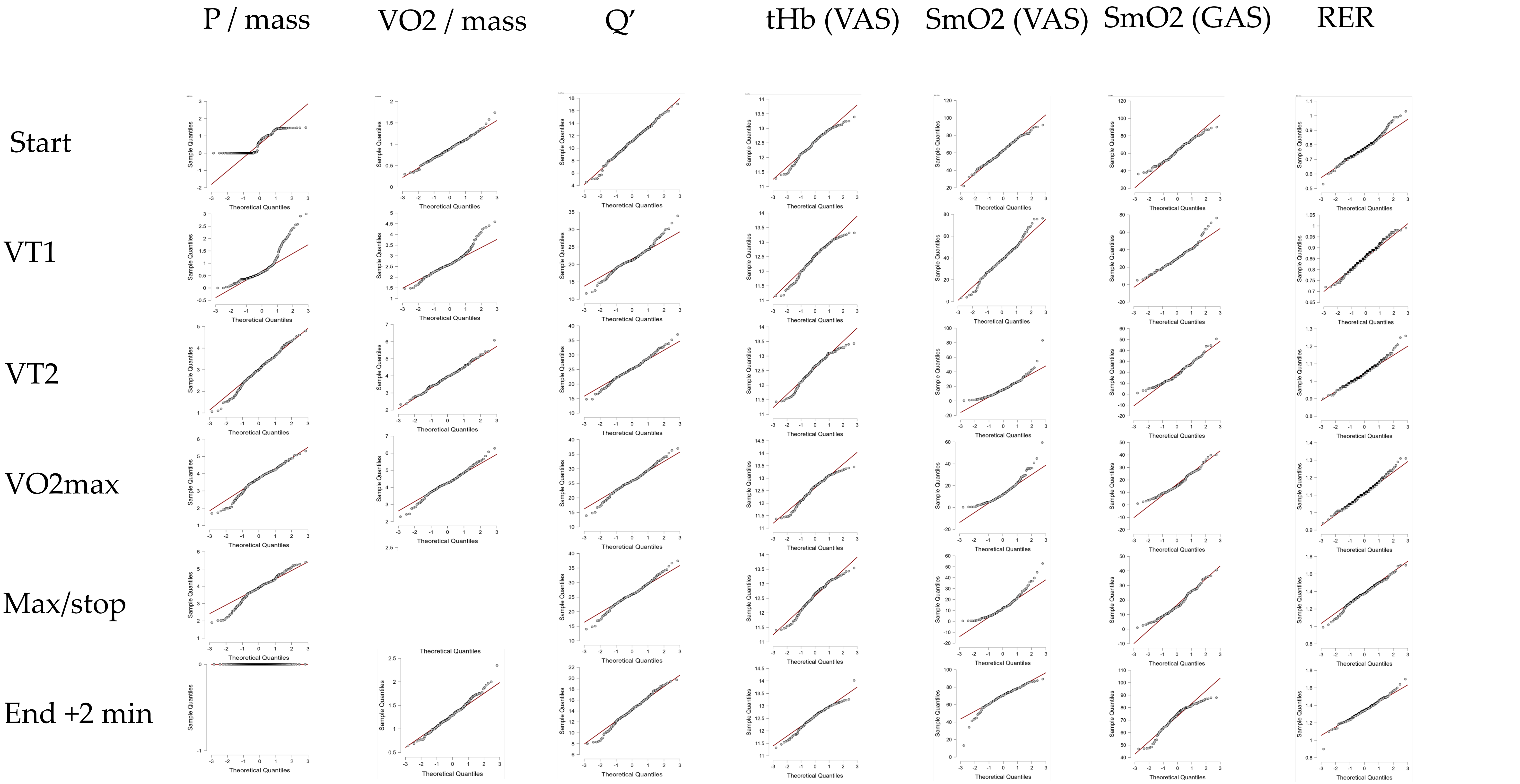

Figure S2. *Q-Q plots of the distribution of residuals for the assessed proxy variables of systemic oxygen transport.* Data were calculated from the available data from the 251 studied subjects at the different intensities (start, VT1, VT2, VO2max,end + 2min). Individual panels at the same vertical height correspond to the intensity given to the far left of the figure. X- and Y-axes refer to the theoretical quantiles and sampled quantiles for the respective proxy variable at a given intensity. Abbreviations: VAS, m. vastus lateralis; GAS, m. gastrocnemius. For further information see the main manuscript.
