# Supplementary material for: Genotypic Influences on Actuators of Aerobic Performance in Tactical Athletes
Source: Genes (Basel). 2024 Nov 28;15(12):1535. doi: 10.3390/genes15121535 (PMC11675622; doi:10.3390/genes15121535)
Supplement: Supplementary file 1 [file genes-15-01535-s001.zip › Figure S3-R1.pdf]

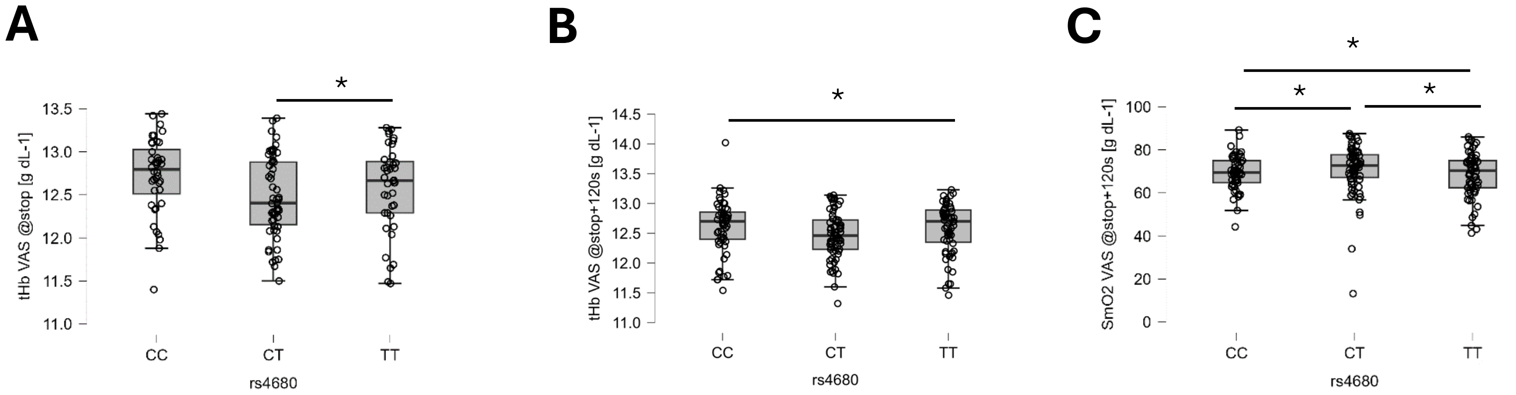

Figure S3: *Genotype effects on recovery of total hemoglobin concentration in vastus lateralis muscle after exhaustive ramped loaded running exercise.* A-C) Box plots with individual values (circles) for the influence of rs4680 on tHb in VAS at the stop of running (A), and 120 seconds after the stop of running (B), as well as on SmO2 in VAS 120 seconds after the stop of running (C). The Y-axes resume the identity of the respective response variable and applicable unit, while the x-axes indicate the respective genotypes of the adressed gene polymorphism. Respective Bayes factors (BF10) for post hoc effects are given as follows: *, 10.0 ≥ BF10 >2.5: **, 30.0 ≥ BF10 >10.0; ***, BF10 > 30.0. Abbreviations: SmO2, muscle oxygen saturation; tHb, total hemoglobin; VT, ventilatory threshold.
