# Supplementary material for: Genotypic Influences on Actuators of Aerobic Performance in Tactical Athletes
Source: Genes (Basel). 2024 Nov 28;15(12):1535. doi: 10.3390/genes15121535 (PMC11675622; doi:10.3390/genes15121535)
Supplement: Supplementary file 1 [file genes-15-01535-s001.zip › Table S1.pdf]

Table S1: *Descriptive Statistics of the assessed proxy variables of systemic oxygen transport.* Data from the 251 subjects studied were assessed regarding aspects that characterize the normal distribution of the proxy variables at the different intensities (start, VT1, VT2, VO2max,end + 2min). See the main manuscript for the abbreviations. SD, standard deviation; SE, standard error. NaN, not applicable as all values were identical.

P / mass	*start*	*VT1*	*VT2*	*VO2max*	*Max/stop*	*End +2min*
missing	13	11	18	11	11	34
Mean	0.60	0.81	2.99	3.64	3.85	0.00
SD	0.57	0.57	0.71	0.70	0.69	0.00
Skewness	0.17	1.57	-0.23	-0.57	-0.56	NaN
SE Skewness	0.16	0.16	0.16	0.16	0.16	0.17
Kurtosis	-1.61	2.20	0.02	0.26	0.39	NaN
SE Kurtosis	0.31	0.31	0.32	0.31	0.31	0.33
Shapiro-Wilk	0.82	0.84	0.99	0.97	0.97	NaN
P Shapiro-Wilk	< .001	< .001	0.15	< .001	< .001	NaN
Minimum	0.00	0.00	1.06	1.70	1.90	0.00
Maximum	1.48	3.00	4.79	5.31	5.40	0.00

VO2 / mass	*start*	*VT1*	*VT2*	*VO2max*	*Max/stop*	*End +2min*
missing	13	11	18	11		34
Mean	11.30	33.79	49.85	53.93		16.54
SD	2.74	5.97	6.78	6.93		2.82
Skewness	0.24	0.80	0.17	0.15		0.14
SE Skewness	0.16	0.16	0.16	0.16		0.17
Kurtosis	0.16	0.94	0.54	0.39		0.74
SE Kurtosis	0.31	0.31	0.32	0.31		0.33
Shapiro-Wilk	0.99	0.96	0.99	0.99		0.99
P Shapiro-Wilk	0.17	< .001	0.26	0.45		0.30
Minimum	4.52	21.20	32.40	35.00		8.32
Maximum	19.27	57.60	76.80	79.00		26.11

Q'	*start*	*VT1*	*VT2*	*VO2max*	*Max/stop*	*End +2min*
missing	15	11	18	11	11	57
Mean	11.07	21.52	25.30	25.88	26.01	14.18
SD	2.28	3.32	3.72	3.84	3.86	2.38
Skewness	-0.17	0.28	0.05	-0.10	-0.09	-0.21
SE Skewness	0.16	0.16	0.16	0.16	0.16	0.18
Kurtosis	0.16	1.40	0.47	0.70	0.75	0.02

SE Kurtosis	0.32	0.31	0.32	0.31	0.31	0.35
Shapiro-Wilk	0.99	0.97	0.99	0.99	0.99	0.99
P Shapiro-Wilk	0.48	< .001	0.33	0.05	0.04	0.23
Minimum	4.56	11.70	14.76	13.91	13.99	8.01
Maximum	17.08	33.91	37.04	36.98	37.47	19.71

tHb (VAS)	*start*	*VT1*	*VT2*	*VO2max*	*Max/stop*	*End +2min*
missing	59	54	71	68	66	64
Mean	12.50	12.46	12.57	12.59	12.57	12.55
SD	0.43	0.49	0.47	0.49	0.48	0.43
Skewness	-0.56	-0.55	-0.51	-0.58	-0.48	-0.38
SE Skewness	0.18	0.17	0.18	0.18	0.18	0.18
Kurtosis	-0.08	-0.27	-0.34	-0.29	-0.38	0.35
SE Kurtosis	0.35	0.35	0.36	0.36	0.36	0.35
Shapiro-Wilk	0.97	0.97	0.97	0.96	0.97	0.98
P Shapiro-Wilk	< .001	< .001	< .001	< .001	< .001	0.00
Minimum	11.28	11.16	11.43	11.37	11.40	11.32
Maximum	13.39	13.32	13.43	13.45	13.54	14.02

SmO2 (VAS)	*start*	*VT1*	*VT2*	*VO2max*	*Max/stop*	*End +2min*
missing	66	67	76	82	70	70
Mean	62.61	38.55	17.00	13.67	13.09	69.48
SD	12.70	13.87	11.16	9.63	9.21	10.38
Skewness	-0.13	0.12	1.72	1.38	1.24	-1.40
SE Skewness	0.18	0.18	0.18	0.19	0.18	0.18
Kurtosis	-0.24	0.55	6.72	2.95	2.12	4.77
SE Kurtosis	0.36	0.36	0.37	0.37	0.36	0.36
Shapiro-Wilk	0.99	0.99	0.90	0.91	0.92	0.92
P Shapiro-Wilk	0.46	0.05	< .001	< .001	< .001	< .001
Minimum	22.00	3.40	0.50	0.20	0.40	13.20
Maximum	91.80	76.30	83.20	59.50	53.00	89.20

SmO2 (GAS)	*start*	*VT1*	*VT2*	*VO2max*	*Max/stop*	*End +2min*
missing	79	80	88	92	84	90
Mean	62.79	30.68	18.79	16.44	16.61	72.82
SD	12.04	12.21	9.25	8.00	8.10	9.67
Skewness	0.02	0.81	0.72	0.65	0.58	-0.86
SE Skewness	0.19	0.19	0.19	0.19	0.19	0.19
Kurtosis	-0.72	1.50	0.39	0.10	-0.19	0.23

SE Kurtosis	0.37	0.37	0.38	0.38	0.37	0.38
Shapiro-Wilk	0.99	0.96	0.96	0.96	0.97	0.93
P Shapiro-Wilk	0.09	< .001	< .001	< .001	< .001	< .001
Minimum	36.30	5.00	1.00	1.00	1.00	46.90
Maximum	90.00	76.30	50.60	40.00	40.50	88.00

RER	*start*	*VT1*	*VT2*	*VO2max*	*Max/stop*	*End +2min*
missing	13.00	11.00	18.00	11.00	11.00	34.00
Mean	0.78	0.86	1.05	1.11	1.38	1.34
SD	0.08	0.06	0.06	0.07	0.13	0.11
Skewness	0.48	-0.05	0.49	0.34	-0.33	-0.02
SE Skewness	0.16	0.16	0.16	0.16	0.16	0.17
Kurtosis	0.93	-0.26	0.70	0.22	0.48	1.26
SE Kurtosis	0.31	0.31	0.32	0.31	0.31	0.33
Shapiro-Wilk	0.98	0.99	0.98	0.99	0.99	0.99
P Shapiro-Wilk	< .001	0.15	0.01	0.08	0.02	0.05
Minimum	0.53	0.72	0.90	0.94	0.99	0.90
Maximum	1.03	0.99	1.26	1.31	1.70	1.70

Lactate					*Max/stop*	*End +5min*
missing					13	13
Mean					11.69	13.31
SD					3.51	3.36
Skewness					0.32	-0.27
SE Skewness					0.16	0.16
Kurtosis					1.08	0.30
SE Kurtosis					0.31	0.31
Shapiro-Wilk					0.98	0.99
P Shapiro-Wilk					0.00	0.43
Minimum					0.50	1.10
Maximum					23.30	22.30
